# Fidelity of a Bacterial DNA Polymerase in Microgravity, a Model for Human Health in Space

**DOI:** 10.3389/fcell.2021.702849

**Published:** 2021-11-29

**Authors:** Aaron H Rosenstein, Virginia K Walker

**Affiliations:** ^1^ Institute of Biomedical Engineering, University of Toronto, Toronto, ON, Canada; ^2^ Department of Biology, Queen’s University, Kingston, ON, Canada

**Keywords:** klenow exonuclease, DNA repair, microgravity, base substitutions, DNA deletions, next generation sequencing, polymerase fidelity, parabolic flight

## Abstract

Long-term space missions will expose crew members, their cells as well as their microbiomes to prolonged periods of microgravity and ionizing radiation, environmental stressors for which almost no earth-based organisms have evolved to survive. Despite the importance of maintaining genomic integrity, the impact of these stresses on DNA polymerase-mediated replication and repair has not been fully explored. DNA polymerase fidelity and replication rates were assayed under conditions of microgravity generated by parabolic flight and compared to earth-like gravity. Upon commencement of a parabolic arc, primed synthetic single-stranded DNA was used as a template for one of two enzymes (Klenow fragment exonuclease+/−; with and without proofreading exonuclease activity, respectively) and were quenched immediately following the 20 s microgravitational period. DNA polymerase error rates were determined with an algorithm developed to identify experimental mutations. In microgravity Klenow exonuclease+ showed a median 1.1-fold per-base decrease in polymerization fidelity for base substitutions when compared to earth-like gravity (*p =* 0.02), but in the absence of proofreading activity, a 2.4-fold decrease was observed (*p =* 1.98 × 10^−11^). Similarly, 1.1-fold and 1.5-fold increases in deletion frequencies in the presence or absence of exonuclease activity (*p =* 1.51 × 10^−7^ and *p =* 8.74 × 10^−13^), respectively, were observed in microgravity compared to controls. The development of this flexible semi-autonomous payload system coupled with genetic and bioinformatic approaches serves as a proof-of-concept for future space health research.

## Introduction

Future long-term space missions may be associated with substantial genomic risks given the prolonged exposure to a lack of substantial gravity (microgravity) and ionizing radiation. While evidence for the ability of ionizing radiation to mutagenize DNA has been investigated, the effects of microgravity on DNA replication and repair of radiation-induced lesions has been less studied. In order to predict the viability of future long-term spaceflight, it is important to understand if microgravity can impact DNA processes and how these dynamics can affect genomic integrity.

The two primary sources of space radiation of concern for upcoming space missions to the Moon and Mars are galactic cosmic rays, ejected from supernovae throughout the universe, and solar particle events emanating from the Sun (Takahashi et al., 2018). Collisions of emitted particles such as neutrons, protons, α and β particles, and especially high-energy/charge particles with DNA are a threat to genomic health. Indeed, cultured rat liver cells on board the International Space Station showed evidence of double-stranded breaks (DSBs) along dense particle tracks consistent with damage due to ionizing radiation damage (Ohnishi et al., 2009). Energized particles can free electrons from DNA nuclei via coulombic interactions or indirectly by collisions with water molecules, forming reactive oxygen species (ROS) (Cannan and Pederson, 2016). Resultant DNA damage includes DSBs, single-stranded breaks (SSBs), crosslinking, depolymerization, base release and base modifications (Pouget et al., 2002; Dextraze et al., 2010; Kennedy, 2014; Santivasi & Xia, 2014).

Countering this DNA damage, however, are the numerous mechanisms which organisms have evolved to repair DNA damage. Mutated bases can be repaired by well-known mechanism such as mismatch, base-excision, and nucleotide-excision repair mechanisms, and DSBs resolved by homologous recombination and non-homologous end-joining (NHEJ), with translesion synthesis important for tolerating pre-existing damage during DNA replication. Nevertheless, exposure to ionizing radiation in space places significant stress on DNA repair and replication pathways. Additionally, there is limited evidence that microgravity can also result in DNA damage via the generation of SSBs/DSBs as well as ROS production (Li et al., 2015). This effect is accompanied by alterations to cytoskeletal proteins, decreased cell adhesion, increased cellular proliferation, and changes in gene expression including p53-mediated apoptotic upregulation linked to cancer pathologies (Battista et al., 2012). Even less elucidated is the interplay of microgravity and radiation on DNA repair with numerous studies presenting puzzling and contradictory results. For example, one space shuttle experiment demonstrated that *Rad54-3* mutant yeast cells, which are temperature-conditional for DSB-repair, had reduced DNA repair capabilities in orbit, but no decrease in DSB resolution was seen after a later flight (Pross et al., 2000). In a second example, cultured cells displayed impaired DSB resolution capabilities after land-based irradiation but no observable DSB repair changes aboard the International Space Station where they would be exposed to both radiation and microgravity (Horneck et al., 1997; Li et al., 2018). Thus questions remain about the effects of microgravity on DNA repair and if it is a concern for future crewed space-travel.

Space-based microgravity, as well as microgravity for ∼20 s intervals during parabolic flight, is termed “real microgravity”, the latter involving placing a payload on a specialized airplane and flying in a parabolic flight-path that induces transient periods of microgravity. Conversely, “simulated microgravity” (SMG), obtained using a 3D clinostat where the rotation of the clinostat negates gravitational force, has contributed to some contradictory results when compared to those obtained in real microgravity (Moreno-Villanueva et al., 2017). Theoretically, space-based testing should most faithfully replicate microgravitational conditions, but it can be logistically challenging and suffer from interference by background radiation. Thus, for DNA repair and replication research, we posit that parabolic flight is a superior alternative to spaceflight studies and more accurately replicates microgravity than SMG.

While distinct cellular mechanisms can repair diverse modes of DNA damage, almost all rely on DNA polymerase enzymes for 5′→3′ templated synthesis of nucleotides to fill in gaps generated by other enzymes. Five polymerase isoforms in prokaryotes (I-V) and at least 15 distinct eukaryotic DNA polymerases (α-ν) as well as telomerase are grouped into 7 families (Garcia-Diaz and Bebenek, 2007). Polymerases have various activities with a generally conserved kinetic mechanism across phylogenies. Polymerases equipped with proofreading capabilities remove mismatched bases in the 3′→5′ direction, and those with nick translation capabilities remove downstream nucleotides in a 5′→3′ fashion. Depending on the presence of these additional activities, polymerases replicate DNA at distinctive rates and with characteristic error frequencies or fidelity. As such, the error rate describes how often base-incorporation errors occur during polymerization of DNA, whether by base substitution, insertion, or deletion. Substitutions may occur due to keto-enol tautomerization or oxidation of DNA bases, resulting in non-canonical base-pairing (Pray, 2008). Insertions and deletions, however, are generally thought to be precipitated by strand-slippage of either the template or nascent strands during replication. A derivative of DNA polymerase I (family A), the large Klenow fragment, lacks 5′→ 3′ exonuclease activity but retains the 3′→5′ proofreading exonuclease domain, and thus is dubbed Klenow (exo+). Klenow (exo-) has neither 5′→3′ or 3′→5′ exonuclease activities due to inactivating mutations (D355A and E357A) in the proofreading domain (Derbyshire et al., 1988). Since this fragment has been widely studied and is readily available in both exonuclease variants, it is considered a model polymerase.

Consistent with their cellular function, DNA polymerases are both highly accurate and rapid in incorporating incoming dNTPs into a growing DNA polymer. For example, the Klenow fragment enters its steady-state phase within 20 s, as pre-steady state elongation occurs within the first 100 msec from reaction initiation (Dahlberg and Benkovic, 1991; Singh et al., 2007). The base substitution error rate is low at ∼1.8 × 10^−5^ for Klenow (exo+) and 4 × 10^−4^ for the exo- enzyme (Bebenek et al., 1990; Lee et al., 2016). In contrast, next-generation sequencing technologies produce errors at frequencies orders of magnitude larger than polymerases (Bebenek et al., 1990; Lee et al., 2016; Potapov and Ong, 2017). To overcome this limitation, unique molecular identifier (UMI) DNA barcodes can permit sequence errors to be identified and deconvoluted from polymerase errors *in silico* (Lee et al., 2016). Although this strategy has not been previously applied to polymerases in microgravity, earlier experiments have suggested that polymerase fidelity is not different in space when compared to earth-like gravity (Ohnishi et al., 2001). Hitherto, however, there has been no investigation of proofreading 3′→5′ exonuclease functionality, nor any characterization of insertion or deletion mutations in space. DNA polymerization reactions in real microgravity can be initiated upon commencement of a parabola and quenched prior to exit from the microgravity phase of the flight trajectory. Since parabolic flights are necessarily time-limited, a modular payload was developed to semi-autonomously conduct experiments. Here we present a novel paradigm for conducting DNA repair and replication experiments in microgravity in order to investigate a central element of the pathway, using a model DNA polymerase, These experiment are timely as astronauts are currently preparing to undertake prolonged exploratory missions where robust polymerase repair activities are essential for survival.

## Materials and Methods

### Single-Stranded DNA Template Design

A 1 kb ssDNA template was designed to have minimal repeats and intra/inter strand reverse-complementarity (both ≤7 nt within 100 bp and ≤5 nt within 25 bp) to avoid secondary structure formation that could complicate downstream polymerization reactions. Approximately 10,000 candidate sequences were generated, and those with ∼50% G + C (guanine and cytosine) content were retained, corresponding to the approximate G + C content of the *E. coli* genome, from which Klenow fragment is derived (Chan et al., 2012). Optimal candidate sequences were identified by their predicted folding energy at 37°C using the MFold server (SantaLucia, 1998). The selected ssDNA template had a G + C% of 50.6% and a theoretical folding energy (∆G) of 15.68 kcal/mol, the highest ∆G of the candidate sequences and much higher than a randomly generated sequence of equal length and G + C% with ∆G≅ −80 kcal/mol. The sequence was dubbed the PolERIS (Polymerase Error Rate In Space) sequence.

### UMI-Barcoded Single-Stranded DNA Template Library Design and Generation

To distinguish microgravity-induced DNA polymerase errors from sequencer-derived errors, 20 nt unique molecular identifiers (UMIs) were incorporated downstream of the polymerization primer binding site on each template molecule (Supplementary Figure S1). This facilitated copying of the UMI to the polymerized strand during each reaction in microgravity and was essential for downstream consensus read generation and subsequent high-accuracy error counting. As such, a protocol using three consecutive rounds of polymerase chain reaction (PCR) and two rounds nucleolytic digestion was developed to generate a template library with diversity of UMIs and high yield and is further documented in Supplementary Materials.

### DNA Polymerization Protocol

The DNA polymerization protocol was designed to simultaneously replicate millions of UMI-barcoded templates within the 20 s microgravity period of each parabola, without any carryover of the reaction into the subsequent pullout period at twice earth gravity. Various reaction parameters were considered and tested in the laboratory including concentrations of ssDNA, enzyme and primers, in addition to an appropriate polymerase inhibitor solution and inactivation temperature. Prior to flight, a 20 μL solution of 1X NEBuffer 2 (New England Biolabs, Ipswich, MA, United States), containing 1.2 pmol of ssDNA template and 100 nM of PolERIS RVS polymerization primer (CGG​AGT​TCA​ATC​GCT​TCG​GCA​ACG) were annealed at 90°C for 1 min, followed by a −0.1°C/sec ramp to 25°C. Template-primer duplexes were then mixed into a 180 μL solution of 200 μM deoxynucleoside triphosphate mixture (dNTPs) and 1X NEBuffer 2.0, yielding a 1X reaction mixture. Enzyme mixtures consisted of 0.5 U/μL of Klenow exonuclease+/- (New England Biolabs) or a no-enzyme control, 1X NEBuffer 2, and 200 μM dNTPs. Inhibitor mixtures consisted of 62.5 mM EDTA in a 1X NEBuffer 2 solution. EDTA was an effective inhibitor due to its ability to rapidly chelate Mg^2+^ ions, essential for both polymerase and 3′→5′ exonuclease activity. To initiate a reaction, 8 μL of each enzyme solution was mixed with 10 μL of reaction mixture and incubated at 37°C. After ∼20 s (depending on the timing of a parabola’s microgravity period, or a corresponding control period), 8 μL of inhibitor solution was immediately injected into each reaction tube and thoroughly mixed, with the reactions also heated to 65°C for 25–90 min, to ensure that all enzymes were irreversibly inactivated.

### Payload

To effectively automate polymerization of DNA templates in microgravity in a repeatable and safe manner, a custom payload for the Canadian National Research Council (NRC) Falcon-20 parabolic flight plane was developed. The payload was designed to perform ancillary functions such as sample storage and incubation, continuous telemetry monitoring of each subsystem, and communication with a laptop computer. The payload comprised a custom-built thermal cycler, robotic pipetting apparatus, control and communication board, on-payload telemetry as well as a block for pre-reaction sample storage. In addition, a housing was built to secure a MyBlock™ (Benchmark Scientific, Edison, NJ, United States) mini digital dry bath heater for sample storage following reactions. All components were housed in a robust aluminum frame and mounted within a Pelican™ (Pelican, Torrance, CA, United States) case customized by the Canadian Space Agency. A camera (Logitech, Lausanne, Switzerland) was installed on the payload to monitor robotic pipette actions while the customized case was closed. All telemetry and control software were developed to wirelessly communicate with the payload, in effect a miniature laboratory, capable of temperature regulated sample incubation, pipetting, thermal cycling, and storing reaction tubes (Supplementary Figure S2).

During payload operation the robotic pipetting assembly was programed to take up 80 μL of each enzyme mixture and three 80 μL aliquots of inhibitor. In-flight, three 0.2 ml reaction mixture tubes were loaded into the thermal cycler, robotic pipette injector needles were immersed in the tubes, and the thermal cycler lid was sealed and set to 37°C. Once the plane’s internal accelerometer read <0.1 G during parabolic flight, manual triggering of the reactions was initiated. The robotic pipette injected 8 μL of the three enzyme mixtures into their respective reaction tubes, with subsequent mixing carried out by a dedicated high-speed mixing pipette. The 8 μL fractions of inhibitor solutions were then injected into each of the reaction tubes when the accelerometer indicated microgravity at >0.1 G, initiated with the manual trigger. Simultaneously, reactions were mixed, and the thermal cycler temperature maintained at 65°C until level flight was regained.

The reaction tubes were removed from the thermal cycler during level flight and placed in the digital dry bath (65°C) until landing. Injection and mixing needles were wiped clean, and 2 μL each of the enzyme and inhibitor mixtures was ejected from the needles to ensure that there would be no cross-contamination from a previous experiment during the next parabola. The mixing pipette tubing was flushed with distilled water followed by the air-pressure generated from rapid depression of a 20 ml syringe. Needles were immersed in distilled water and wiped again. The enzyme and inhibitor fluids were then recessed into the pipette tubing such that a 2 μL air bubble was retained between the meniscus of each fluid and the tip of its respective needle. Once the next set of reaction mixture tubes were inserted, the air cushion prevented premature initiation and inhibition of the next reaction prior to appropriate microgravity conditions. Reactions were performed simultaneously for each enzyme mixture. Two reactions, corresponding to Klenow (exo+) and Klenow (exo-) respectively, were performed during level flight to act as earth-like gravity (1 G) controls. The payload was designed to execute a maximum of 6 independent reactions in microgravity, but due to logistical constraints during the flight, only two reactions were conducted in microgravity, the first of which was used for analysis.

### Sample Post-Flight Processing

After landing, samples were removed from the plane, immediately frozen at −20°C and returned to the laboratory where they remained frozen until sample post-flight processing. Prior to sequencing library preparation, all samples were digested with Exonuclease VII (New England Biolabs) to remove ssDNA tails from the partially polymerized DNA species in order to yield trimmed dsDNA molecules containing only polymerized DNA. Following this, the NEBNext Ultra II Library Preparation kit (New England Biolabs) was used to prepare samples for sequencing on the NovaSeq 6,000™ (Illumina, San Diego, CA). Further information regarding the post-flight processing protocol is documented in Supplementary Materials.

### Bioinformatic Analysis

Polymerization reactions in both 1 G and microgravity (μG) were conducted in flight for both Klenow (exo+) and Klenow (exo-) DNA polymerases, with sample identities designated as 1 G+/1 G− and μG+/μG− respectively where + and–referred to the presence or absence of exonuclease activity. For each sample, each of the triplicate libraries were assessed separately in the event of experimental pipeline error. Initial base-calling and dual index barcode demultiplexing operations were performed using *bcl2fastq2 v2.20* by the sequencing vendor (TCAG, Toronto, ON, Canada).

Alignment of paired end reads from each sample was completed using Bowtie2 (Langmead and Salzberg, 2012). Alignment was completed using the “very sensitive” parameters, as these most effectively called single-base errors on the small reference sequence. The output sequence alignment map/format (SAM) files were then read into R, and alignment flags from Bowtie2 were parsed to identify which reads represented template versus polymerized strands. The code for conducting this process, and the rest of this section is included in Supplementary Data.

UMIs were identified for each sequence read with a custom program written to parse the alignment data for each read in order to extract its UMI. As well, the Q-scores for each base in the UMI were identified and the probability of UMI misidentification was calculated as:

$$P=1-\overset{20}{\underset{i=1}{\prod }}\left(1-10^{\frac{-Q_{i}}{10}}\right)$$

Where *P* represents the probability of an incorrect base call for each Q score. Following this, a deduplicated list of UMIs was tabulated, and a radix tree was generated using these values. In order to bin each read with other reads based on their common UMI, a single numerical identifier was assigned to each distinct UMI. Each read was then grouped with others that shared its respective identifier using a radix tree, facilitating rapid searching and read binning by UMI. To make efficient use of the data collected, reads were selectively re-binned when their UMIs were within one base mismatch of exactly one pre-existing UMI bin. However, this process only occurred if the origin UMI bin had a single member read mate-pair, and the destination bin had more than 5 mate-pairs. Following re-binning, all reads within UMI bins with <6 read mate-pairs were discarded. Reads were then grouped based on the results of the UMI re-binning process. Bins without adequate numbers of both template and polymerized strand reads were discarded, as strand comparison was not possible in this scenario. UMI binning, re-binning, and successive steps in analysis are depicted in Supplementary Figure S3.

Errors were called sequentially by UMI bin. This involved stacking template reads to determine the consensus base at each locus and comparing it to the consensus base representing the stacked polymerized-strand reads. For each base-per-base comparison, whether it was an error or not, the probability of a false consensus, and thus an incorrect error call, was identified. This included computing the probability of every permutation of correct/incorrect bases at a given locus that resulted in a simple majority of bases being equal to the pre-determined consensus base, for template and replicated strands, respectively. Each base was scaled based on the probability of UMI inclusion, thus accounting for a false addition to a given UMI bin.

To identify the processing error (P_E_) for a given base call at a defined locus, on either the template or replicated strand, sequencing quality, consensus calling, and UMI misidentification were represented mathematically. The integer *m* was taken to represent the number of defined bases at a specific locus for a given UMI. If *m* was greater than 12 for a given UMI bin, a random subset of 12 reads was taken for P_E_ calculation as this reduced the computational complexity of this segment of the analysis. As such, if $n=\overset{m}{\underset{i=1}{\sum }}\left(\begin{array}{c} m\\ i \end{array}\right)=2^{m}-1$ , then $P_{m\;\times \;n}$ represented a matrix of all row-based combinations of reads for a given *m. N* then was taken to represent the set of row indices of *P* where row sums were greater than *m/2,* thus representing scenarios where a simple majority existed for a given base. As well, *q* represented the vector of error probabilities for each base on each read at a given locus. The probability of incorrect UMI binning corresponding to each read was represented as *b*. Finally, *e* represented a logical vector indicating whether each base on each read at a given locus was in error, not matching the pre-defined consensus base for that locus in the UMI bin. Therefore, the P_E_ for each locus was calculated as:

$$P_{E}=\underset{i\;\in \;N}{\sum }\overset{m}{\underset{j=1}{\prod }}\left|e_{j}-\left|P_{ij}-1+\left(1-b_{j}\right)\left(1-q_{j}\right)\right|\right|$$

To aid in constraining the analysis to reads that were viable for accurate error calling, only reads with template:polymerized strand ratios ranging from 1:3 to 3:1 were identified. As well, for any locus within a read, at least 6 reads were required per template and per replicated strand for error calling. Therefore, output from these analyses were binary values indicating either error or no error, with corresponding P_E_ for such assertions. The expected error of each base was thus calculated as $\left|e-P_{E}\right|$ . Expectations were kept only for bases where the P_E_ fell below a determined threshold so as not to skew final error rate determinations towards low consensus reads with poor quality.

Error rates were calculated independently for each base and each unique base substitution, respectively. The only retained error-rate determinations for bases in the template sequence were those that exhibited sufficiently deep coverage to yield meaningful results (the first 175 bp following the barcode). As a result of inevitable sample degradation from oxidative damage following flight and during sample preparation, 8-oxoguanine mutations induced G → T (guanine to thymine transversion) mutations on template strands and polymerized strands that were extrinsic to those introduced by polymerization reactions during flight. Because polymerized strand sequences were viewed in reference to the template sequence, G → T mutations appeared as C → A (cytosine to adenine transversion) mutations in these sequences. Such artifacts have been previously recorded in deep-sequencing experiments, and must be compensated for in a high-sensitivity experiments (Costello et al., 2013). While the removal of falsely templated strands was conducted by selecting only template reads that matched the pre-defined template sequence, deconvolution of these mutations from polymerized strand reads required filtering outlier loci which were highly susceptible to guanine oxidation. As such, analysis continued using only a C → A mutation rate less than 5 × 10^−4^, which captured the central tendency of this specific base substitution rate without spurious mutation introduction. For base substitutions, data was sectioned at successive levels of specificity. Comparisons for each enzyme overall, by template base as well as by specific substitution error, were undertaken. Furthermore, grouping of error rates by di- and trinucleotide was conducted. Information regarding deletions was collected for each locus in the template sequence and tabulated independently based on the identity of the inserted/deleted base, as well as its preceding nucleotide.

### Statistical Analysis

Pairwise analyses were completed between in-flight 1 G+/1 G− and μG+/μG− test conditions. For each locus on the template, the median error rate across the three triplicate samples for each test condition was used for analysis as this dissipated possible variability due to library preparation. Regardless, minimal to no variability was observed between triplicates for any test condition. Calculated error rates for most bases in the template were centered around a median value, while an elongated tail represented specific loci where the error rate was substantially higher than the central tendency for that base. While the mean error rate for a given base intuitively represents the frequency of a given error in a population of templates, it is highly weighted by the small number of outlier loci. However, the median error rate represents the most likely discrete error rate value that a base at a given loci can possess. As a result, Wilcoxon-Mann-Whitney tests were used for the comparison of the skewed substitution and deletion mutation distributions. All pair-wise comparisons were conducted in a two-tailed manner, with a standard α threshold of 0.05 to indicate significance. While the paired version of this test (the Wilcoxon signed-rank test) was indicative of locus-wise alterations in median error rate, the unpaired version (Mann-Whitney U test) was indicative of a global median change in error rate irrespective of sequence context. As such, paired tests were completed when permissible to give more direct statistical interpretation of the data, but when more granular comparisons of error rate were made, such as substitution-wise interrogation, only unpaired tests were a viable means of determining statistical significance. In all tests completed, mean imputation of error rate on a per-locus basis was utilized in order to compensate for missing values in the data. While relatively sparse, error rate calculations for specific substitutions or deletions at defined loci which equated to zero were replaced with imputed values. This was essential to maintaining a balanced error rate determination such that loci with missing values were not weighted less than loci with extant values in triplicate. The code for this section is included in Supplementary Data S2.

## Results

### Flight

**FIGURE 1:**
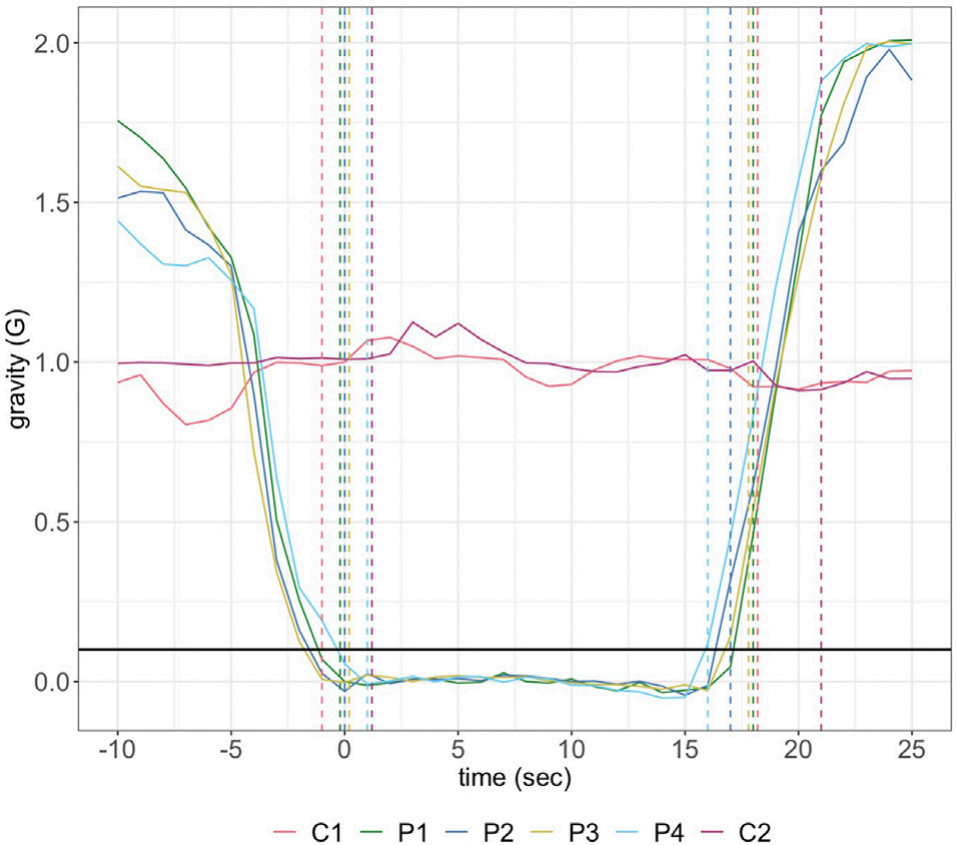
Gravitational load over the time-course of the control (C1, C2) and parabolic (P1–P4) reactions completed during flight. The first and second sets of dashed lines represent the start and end of each reaction, respectively. The reaction initiation threshold of 0.1 G is shown in black across the period.

On May 22nd^,^ 2019, the payload was aboard “Research One” from the NRC Flight Research Laboratory at Ottawa International Airport. There were two control (1 G) experiments in-flight. Due to logistical concerns during flight only the first parabola was used as a data-source for μG experiments, corresponding to the first in-flight 1 G reaction. The first parabola followed the same gravitational load over time as seen for all four parabolas, indicating its suitability for experimental analysis (Figure 1).

### Sequence Analysis and Single Nucleotide Error-Calling

As determined by Bowtie2, reads from each sample aligned >99.5% of the time to the reference sequence, indicating minimal contamination. Bowtie2 alignment also generated polymerization product lengths for each read mate-pair (Supplementary Figure S4). Samples collected in microgravity were denoted as μG+ or μG− depending on whether Klenow (exonuclease+) or Klenow (exonuclease−) were used, whilst earth-like gravity controls were denoted as 1 G+ or 1 G− in a similar fashion.

To integrate the processing error (P_E_) calculation into the error rate analysis, the median expected error values for each sample were plotted for successive P_E_ thresholds and separated by test condition and base identity (Supplementary Figure S5A). A P_E_ threshold of ≤1 × 10^−5^ was chosen since after this point no substantial decrease in overall error rate was observed. The expected error was calculated for each nucleotide under each condition, and although these rates were generally similar under different gravitational conditions, μG− samples showed a slightly higher error rate for thymine bases (Supplementary Figure S5B).

**FIGURE 2:**
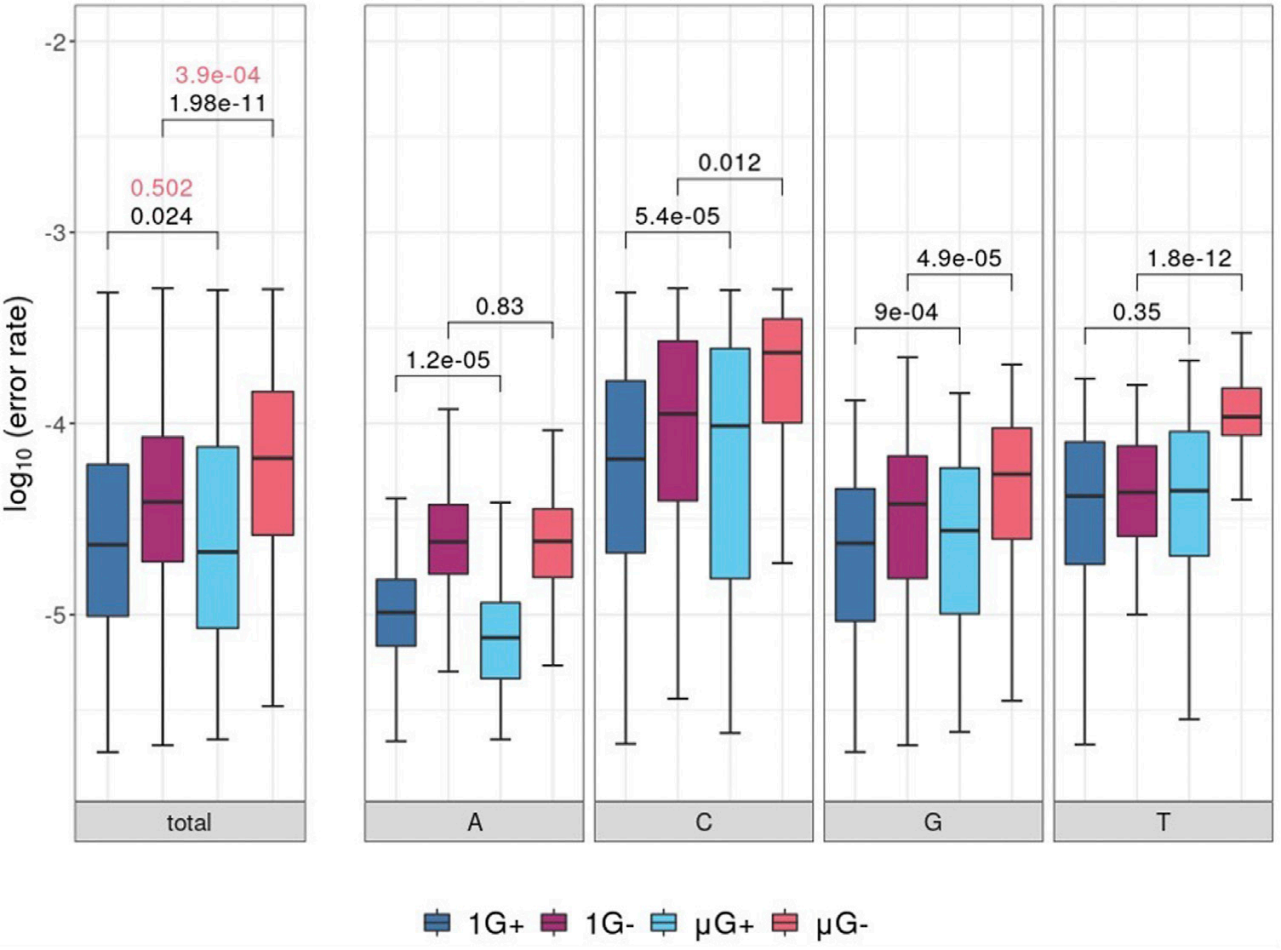
Boxplots of locus-scale polymerase error rate distributions, with different samples showing microgravity (μG) or earth-like gravity (1 G) with + and – referring to the presence or absence of exonuclease activity in the Klenow fragment polymerase, respectively. Significance values of statistical comparisons, which were calculated by Wilcoxon signed-rank tests were plotted in black, while those made using Mann-Whitney U tests were plotted in red. **(A)** Boxplots for each test condition. **(B)** Boxplot of polymerase error rate distributions for each test condition, separated by template nucleotide either adenine (A), cytosine (C), guanine (G) or thymine (T) listed below the clustered boxplots.

Following removal of low-quality bases (either by the P_E_ or from C→A mutations), comparisons of template locus-wise error rate distributions were plotted and separated by nucleotide identity (Figure 2). While there was no significant difference in median error rate between μG+ and 1G+ (when tested using the unpaired Mann-Whitney U test, fidelity was significantly decreased in μG− compared to 1 G- (*p =* 3.9 × 10^−4^). A significant mean 1.1-fold increase in base-wise error rates were observed between 1 G+ and μG+ (*p =* 0.024) in addition to a 2.4-fold increase between 1 G− and μG− (*p =* 1.98 × 10^−11^) when assessed using the Wilcoxon-signed rank test. The increase in substitution error rates in μG ranged up to 3.4 and 27.5-fold higher than 1 G for Klenow (exo+) and (exo−) respectively when assessed in a pairwise fashion. Thus, while overall median error rate was not significantly increased in Klenow (exo+), paired locus-wise assessment of median error rate was statistically significant. This result then prompted analyses of base-wise error rates using a paired test methodology. While Klenow (exo-) introduced significantly more errors on C, G, and T template bases in microgravity (*p* = 0.012, 4.9 × 10^−5^ and 1.8 × 10^−12^ respectively), there was no significant alteration in median error rate for A template bases. C and G template bases were significantly more prone to substitution errors in microgravity when polymerized by Klenow (exo+) (*p* = 5.4 × 10^−5^ and 9 × 10^−4^ respectively), and conversely, polymerization on A was more accurate in μG for this enzyme (*p =* 1.2 × 10^−5^).

**FIGURE 3:**
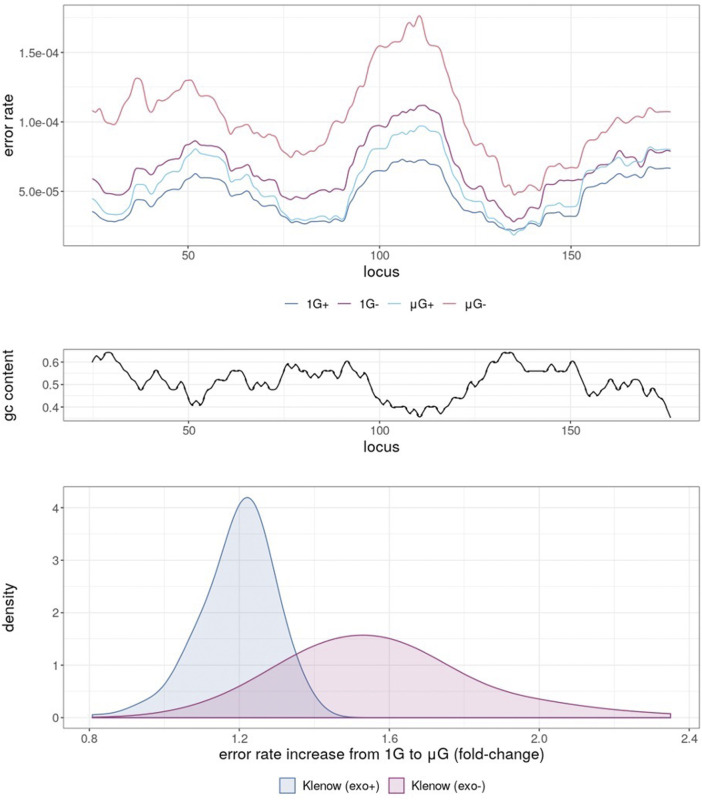
Simple moving average of polymerase error rate across 25 nt windows of template under different experimental conditions, either earth-like gravity (1G) or microgravity (μG) with + and – referring to the presence or absence of exonuclease activity in the Klenow fragment polymerase. **(A)** The mean error rates of each test sample for each 25 nt window moving across the template, such that at locus = 25, error rate was the mean of loci 1–25, with the experimental conditions shown as colored lines corresponding to 1 G+ (blue), 1 G− (mauve), µG+ (turquoise) and µG- (orange). **(B)** G + C% (gc content) in 25 nt windows sliding across the template sequence. **(C)** Density plot of mean error-rate increases in microgravity for Klenow (exo+) and Klenow (exo-) for each 25 nt window along the template.

Mapping of error rates by simple moving average with a period of 10 nt across the template revealed obvious variations depending on the sample sequence (Figure 3A). 1 G+ exhibited the lowest averaged error rate across all template loci, with μG+ tracking closely, but with error rates generally shifted marginally higher and modulated based on template region. Predictably, further error enrichment from Klenow (exo-) samples was generally observed across the template. Under 1 G conditions the absence of proofreading activity resulted in localized mean error rates across a 25 bp sliding window up to 1.12 × 10^−4^ compared to 7.34 × 10^−4^ for Klenow (exo+), again depending upon position. Furthermore, the mean error rate moving across the template was anti-correlated with GC-content (Figure 3B), with Pearson correlation coefficients of −0.82, −0.81, −0.84, and −0.72 for 1 G+, 1 G−, μG+, and μG− samples, respectively. Notably, μG- samples showed consistently higher localized mean error rates across the template than their 1 G counterparts, ranging up to 1.4-fold for Klenow (exo+) and 2.4-fold for Klenow (exo-) depending on template locus (Figure 3C).

**FIGURE 4:**
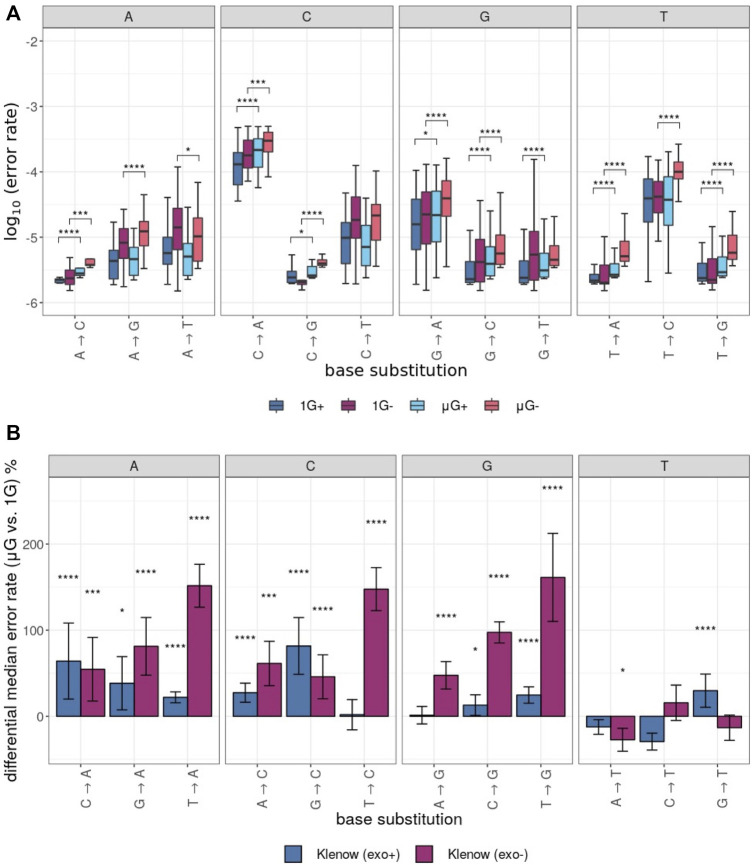
Polymerase error rates interrogated by base substitution identity, including those exposed to either earth-like gravity (1 G) or microgravity (μG) conditions with + and – referring to the presence or absence of exonuclease proof-reading activity in the Klenow fragment polymerase, respectively. Mann-Whitney U test *p*-value symbols are plotted above each boxplot for only those comparisons that were significantly different with *p* < 0.05 = *, *p* < 0.01 = **, *p* < 0.001 = ***, *p* < 0.0001 = ****. X → Y substitutions (where X and Y symbolize either C, G, T or A nucleotides) represent replacement of X with Y on the template sequence. **(A)** Boxplots of the log_10_ error rates by sample and base substitution identity with individual clusters representing the identity of the template base. For each experimental condition, boxes are colored as noted in Figure 3. **(B).** Differential median error rate percentage between gravitational conditions (1 G vs. µG) for each unique base substitution with individual clusters representing the identity of the substituted base. The superimposed error bars represent the standard deviation of each group.

The error rates for each unique base substitution error were also determined for each test sample (Figure 4A). This yielded further granularity and insight into the observed differences in error rate between gravitational conditions (Figure 2B). In general, the directionality of error rate differences, irrespective of the two gravitational conditions, was conserved across the 12 possible assessed substitutions. Furthermore, grouping of error rates by the identity of the substituted base revealed a general trend of the percent difference in median error rate between gravitational conditions (Figure 4B). While substitutions from T templates by Klenow (exo-) were consistently more frequent in μG than in 1 G, mutations from A, C and G resulting in replacement with T were generally less frequent in microgravity, or in two cases more frequent by a small margin (Figure 4B).

### Dinucleotide, Trinucleotide, and Deletion Errors

**FIGURE 5:**
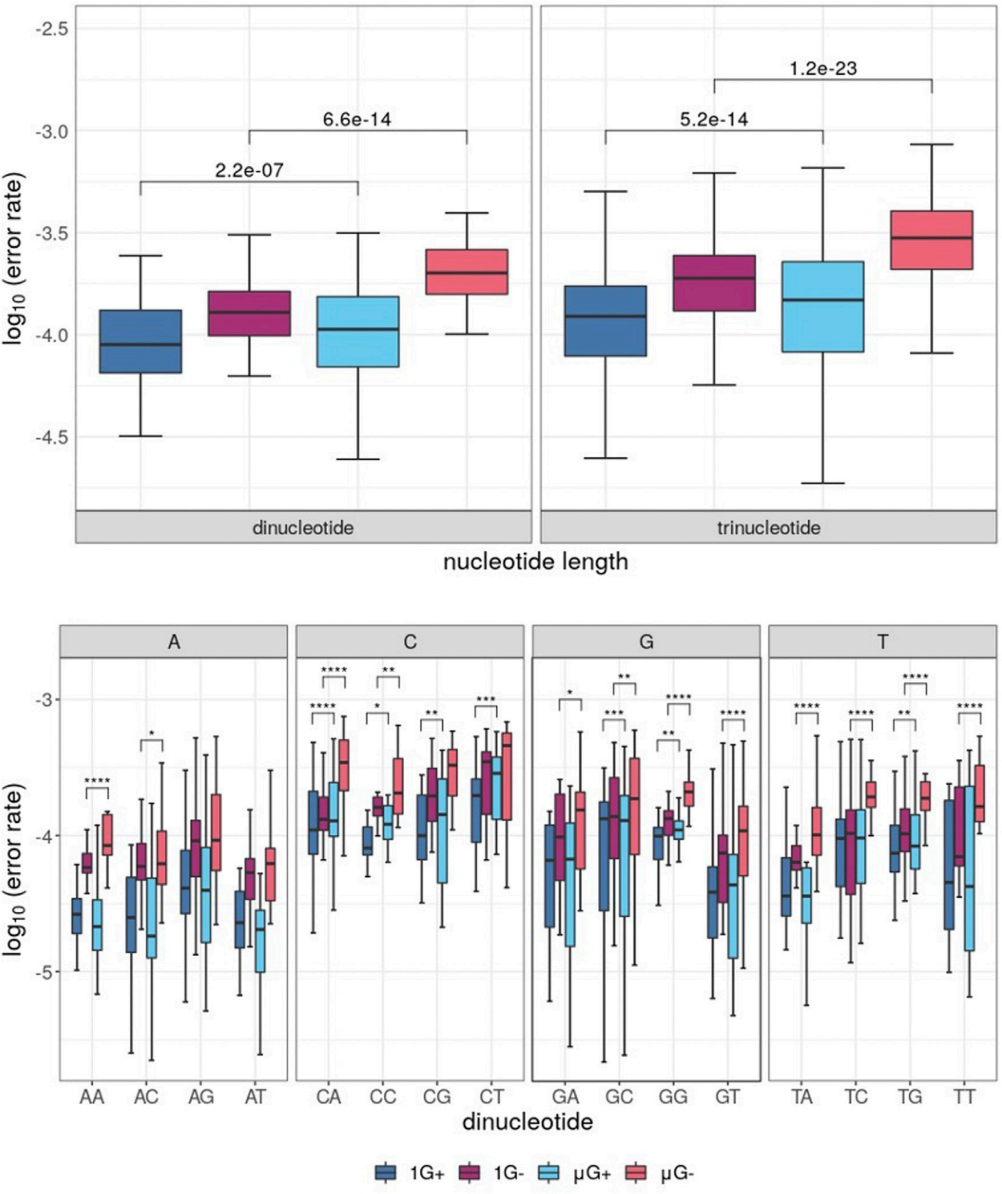
Boxplots of error rates by di- and trinucleotides for each sample under conditions of either earth-like gravity (1 G) or microgravity (μG) with + and – referring to the presence or absence of exonuclease activity in the Klenow fragment polymerase, with boxes colored according to Figure 3. Wilcoxon signed-rank test *p*-value symbols are shown plotted for each significant comparison (*p* < 0.05 = *, *p* < 0.01 = **, *p* < 0.001 = ***, *p* < 0.0001 = ****). **(A)** Boxplot of substitution errors (log_10_) for di- and trinucleotides for each test sample. **(B)** Boxplots of dinucleotide error rates grouped by dinucleotide identity, clustered by the identity of the first template base for a given dinucleotide pair.

When grouped by di-and trinucleotide identity, analysis of substitution errors yielded similar results to those obtained from single-base analysis. Indeed, μG- samples showed the highest error rate irrespective of the analysis (Figure 5A). Thus, both Klenow (exo+) and Klenow (exo-) were significantly more error-prone in μG than 1 G when analyzed by the distribution of median errors when grouped by dinucleotide identity (*p =* 2.2 × 10^−7^ and 6.6 × 10^−14^, respectively). A similar effect was observed when error rates were normalized for trinucleotide identity, but the magnitude of significance values was increased (*p =* 5.2 x 10^−14^ and 1.2 × 10^−23^, respectively). Furthermore, when the substitution error rate was assessed by individual dinucleotides, errors were generally more dependent on the first base in a given dinucleotide than the second (Figure 5B). As might be expected given the propensity for more frequent single-nucleotide errors associated with C, G and T in microgravity, CN, GN, and TN (where N = any base) template nucleotides were generally more error-prone in μG compared to 1G for both enzymes by a statistically significant margin. Similarly, only two significant comparisons were identified between gravitational conditions for AN dinucleotides, both of which were associated with Klenow (exo-).

**FIGURE 6:**
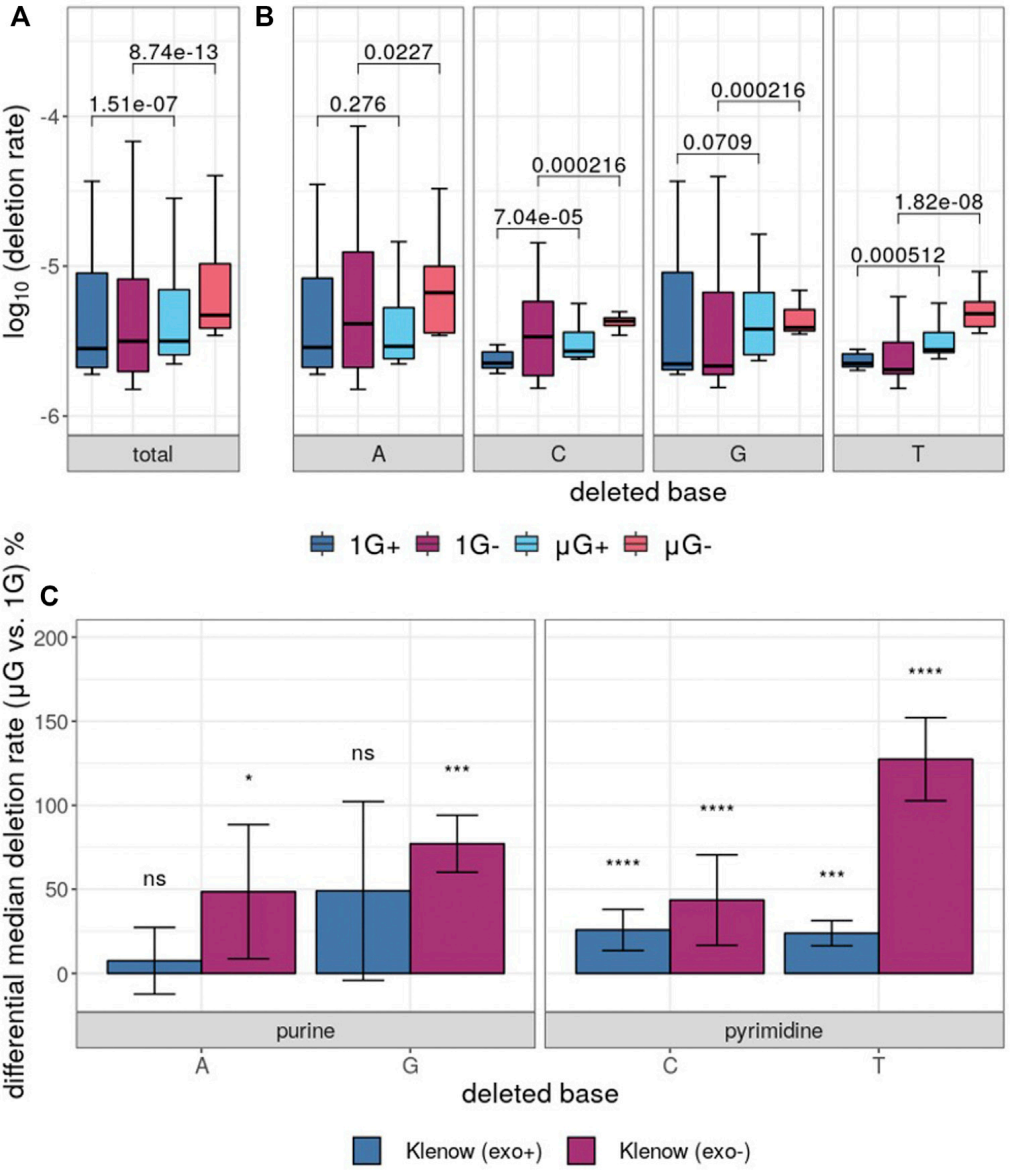
Base deletion error rates in either earth-like gravity (1G) or microgravity (μG) from Klenow fragment polymerase polymerization with (+) or without (−) exonuclease activity. Mann-Whitney U test *p*-value symbols are shown plotted for all comparisons, while significance symbols are shown for significant comparison only (*p* < 0.05 = *, *p* < 0.01 = **, *p* < 0.001 = ***, *p* < 0.0001 = ****). **(A)** Boxplots showing the overall deletion error rate (log_10_) for each test condition and colored according to Figure 3. **(B)** Deletion error rate for each test condition grouped by deleted base, with the boxes colored as indicated. **(C)** Differential percentage deletion error rate for each base, separated by purine versus pyrimidine bases. Bar-plot error bars in (b) and (c) represent the standard deviation of each group.

While base insertions were quantifiable by the deep-sequencing analysis pipeline employed here, these mutations occurred at too low a frequency for statistical analysis with any substantial power (not shown), but investigation of differential base deletion rates between the two gravitational conditions was statistically insightful (Figure 6A,B). There was a significantly higher deletion rate in microgravity for both Klenow (exo+) and Klenow (exo-) samples (*p* = 1.5 × 10^−7^ and 8.7 × 10^−13^, respectively) with effect sizes of 1.1- and 1.5-fold, respectively. Significance was calculated by Mann-Whitney U test due to the relative sparsity of this dataset due to low deletion frequencies, prohibiting the use paired testing methodologies such as the Wilcoxon signed-rank test. Deletion of pyrimidine bases occurred at a significantly higher rate in μG compared to 1 G, irrespective of proofreading activity (Figure 6B,C). While deletion of purines occurred at an overall rate that was generally higher than pyrimidine deletions, only Klenow (exo-) was susceptible to increased deletion rate due to microgravity by a significant margin for these bases. Results from this section, as well as for single-nucleotide errors, are summarized in Table 1.

**TABLE 1:** Summary of error rates including overall substitution errors, base-wise substitutions distinguished by the template nucleotide either adenine (A), cytosine (C), guanine (G) or thymine (T), di- and trinucleotides, overall deletions, and base-wise deletions grouped by individual template nucleotides (A, C, G or T) for each sample under conditions of either earth-like gravity (1 G) or microgravity (μG) in the presence or absence of the Klenow fragment exonuclease activity (exo + or exo-, respectively), with statistical significance calculated using Wilcoxon signed-rank tests or Mann-Whitney U tests (M-Whitney).

	Klenow variant	Error rate		Significance	
		1G	μG	Wilcoxon	M-Whitney
Overall substitution	exo +	2.07E-05	1.83E-05	0.024	0.502
	exo −	3.68E-05	7.51E-05	1.98E-11	0.00039
Base-wise substitution				Wilcoxon	
A	exo +	1.00E-05	7.01E-06	1.20E-05 ***	
	exo −	2.36E-05	2.39E-05	5.40E-05 ***	
C	exo +	6.34E-05	9.58E-05	9.00E-04 **	
	exo −	1.11E-04	2.32E-04	0.35	
G	exo +	2.14E-05	2.29E-05	0.83	
	exo −	3.78E-05	5.36E-05	0.012 *	
T	exo +	4.13E-05	4.29E-05	4.90E-05 ***	
	exo −	4.29E-05	1.08E-04	1.80E-12 ***	
Dinucleotide substitution	exo +	8.95E-05	1.06E-04	2.20E-07 ***	
	exo −	1.29E-04	2.01E-04	6.60E-14 ***	
Trinucleotide substitution	exo +	1.23E-04	1.44E-04	5.20E-14 ***	
	exo −	1.89E-04	2.98E-04	1.20E-23 ***	
Overall deletion	exo +	2.82E-06	3.16E-06	1.51E-07 ***	
	exo −	3.16E-06	4.71E-06	8.74E-13 ***	
Base-wise deletion					
A	exo +	2.87E-06	2.92E-06	0.276	
	exo −	4.13E-06	6.65E-06	0.0227 *	
C	exo +	2.26E-06	2.71E-06	7.04E-05 ***	
	exo −	3.38E-06	4.31E-06	0.000216 **	
G	exo +	2.23E-06	3.80E-06	0.0709	
	exo −	2.16E-06	3.90E-06	0.000216 **	
T	exo +	2.25E-06	2.77E-06	0.000512 **	
	exo -	2.04E-06	4.82E-06	1.82E-08 ***	

Significance comparisons are indicated as follows: * = p < 0.05, ** = p < 0.01, *** = p < 0.001 = ***.

## Discussion

### Payload Performance

The payload successfully performed in the NRC Flight Research Lab parabolic mission. The data from the first 1G control and μG parabola were interrogated, and this portion of the flight obtained by the Falcon-20 pilots was both accurate and precise (Figure 1). Initiating experiments at a precise timepoint on a lab-top computer was a challenge during weightlessness. During an initial test flight (not shown), the transition from μG to up to 2 G resulted in unplugging of the USB communication cable between the payload and computer, and in consequence wireless communication technologies such as WiFi and Bluetooth were deemed more dependable during flight conditions. Even reaching and operating a laptop computer trackpad was challenging in microgravity, and thus a custom trigger handle was 3D printed, for easy and dependable initiation of reactions without requiring full range of motion during μG flight. For the data presented here, the enzyme injections occurred within the planned 0.1 G threshold with the inhibitor injections completed at the ∼0.1 G threshold.

Determination of polymerized template lengths from triplicate sequencing libraries revealed that most were ∼200 bp in length, although the 1 G- libraries were ∼50 bp shorter (Supplementary Figure S4). It is possible that this could represent stochastic variation as both the payload and post-processing exonuclease digestion protocol using agarose gel electrophoresis (not shown) of each test sample did not yield substantial product length variation. The protocols were not designed for precision in the measurement of this parameter, but template lengths were suitably long (∼175 bp) and most base processing errors were sufficiently low such that sample size for error calling was maximized while decreasing the propensity for errors introduced by any low-quality consensus reads. Inspection of the data allowed the qualitative selection of a P_E_ threshold of 5, and this false positive rate threshold held for the error rate of each individual base and indeed did not decrease substantially for lower P_E_ cut-offs (Supplementary Figure S5).

### Microgravity Induces DNA Polymerization Errors

When the error rates of both Klenow (exo-) and Klenow (exo+) were compared between gravitational conditions, a clear negative impact of microgravity was observed. Evidence for increased error rates comprised every measure used, including total substitution errors, most nucleotide-specific errors, averaged error rate moving across the template, the majority of specific substitution errors, dinucleotide and trinucleotide errors and most base deletions (Figures 2–6). Thus, this data is congruent with earlier findings of reduced DNA repair capacity in microgravity, such as observable increases of SSBs in human lymphocytes aboard the International Space Station (Battista et al., 2012). Even more relevant may be the DSBs and base modifications that were generated in SMG-cultured mouse stem cells, which were DNA damage-response deficient (Li et al., 2015), and thus conceptually similar to the Klenow (exo-) enzyme used in this experiment.

Taken together, the results present strong evidence that microgravity acts as a DNA stressor, although the mechanism remains unknown. While the 3′→ 5′ exonuclease domain of Klenow (exo+) appeared to confer a partial ability to “rescue” this polymerase from the microgravity-induced decrease in fidelity, this effect was marginal and by no means neutralized the consequences of microgravity (Figure 3C). As such, it may be the case that regardless of the cause of an increased propensity for polymerization errors observed in microgravity, exonuclease activity is either insufficient in ameliorating this effect or could be deficient in repair in microgravity as well. Certainly, there appeared to be some base-specificity of altered polymerization fidelity in microgravity. Errors generated by inappropriate placement of T were outliers in that they were not subject to the same increase seen with other nucleotides in μG- samples. This may suggest that topological and steric properties of the majority of individual bases (either as residues on the template strand or as the complimentary incoming dNTPs bound to the fingers domain of the polymerase) resulted in enzyme-substrate conformational changes leading to the elevation of most non-canonical base-pairing arrangements in microgravity. Another possible explanation may be related to the lower convective flow in microgravity (Todd, 1989). This could alter local dNTP concentrations surrounding the binding pocket in the polymerase, which can alter polymerization fidelity (Eckert and Kunkel, 1991). Alternatively, μG conditions could alter polymerase conformation and thus impact enzyme activity. Since these suggestions involve structure-function changes in the binding of the polymerase to its substrate, further investigation such as crystallization of the Klenow (exo-) protein in microgravity may be warranted, with dNTP and with other nucleotides. It is also prudent to note that the effects observed here may not be applicable to all DNA polymerases, as proofreading capacity is not consistent both within and between polymerase families.

No matter the mechanism, microgravity DNA polymerization occurred with reduced fidelity, which may explain, in part, the possible synergism that appears to exist between microgravity and radiation, which has far harsher effects on DNA. Our results differ from a previous ground-based investigation in that an alteration in polymerization fidelity was identified in both substitution and deletion mutation rates, and in a non-proofreading polymerase (Potapov and Ong, 2017). As a consequence, a broader interest in enzyme kinetics in microgravity is well warranted. While the polymerases analyzed here may not pose a direct threat to genomic integrity in space, apart from microbiome-associated polymerases, eukaryotic repair enzymes may also be vulnerable to microgravity-mediated altered activities. It may be the case that other polymerases exhibit a more substantial reduction in fidelity in microgravity, or that other enzymes which may exhibit reduced activity in microgravity may further hinder radiotolerance in space. This hypothesis may therefore explain higher order cellular and physiological dysfunction in microgravity and warrants further investigation.

### Klenow Exo+ vs. Exo- Polymerase Comparisons

Although there were no differences in the median mutation rate in μG+ samples compared to 1 G+ samples in overall substitutions (unpaired Mann-Whitney U test), analysis with the Wilcoxon signed-rank test comparing median error rates for each locus in a pair-wise fashion showed a significant effect for both enzymes (Figure 3A). The distribution of fold-increases in error rates across 25 nt windows of the template was smaller for Klenow (exo+) than Klenow (exo-) indicating that polymerization errors in microgravity can be only partially abrogated by the exonuclease domain that can repair base incorporation errors (Figure 3C). Notably, this increased burden on repair machinery in the μG+ samples do not appear to significantly decelerate DNA polymerization since the product length distributions were similar between 1 G+ and μG+ samples (Figures 2–Figures 4). Base-wise substitution error rate analysis revealed that repair fidelity decreases on templated C, G, and T bases were significant for both enzymes with the exception of T-templated polymerization by Klenow (exo+). Conversely, polymerization appeared more accurate for A bases in microgravity (Figures 2B–Figures 6B). We can only speculate that similar to our previous suggestion that the increased fidelity of polymerization of A-templated loci may indicate that microgravity can actually improve the ability of a perhaps slightly conformationally changed DNA polymerase to discern correct base pairing opposite A but not with the other three bases.

Previously, Klenow (exo+) and (exo-) have been documented with error rates of ∼1.8 × 10^−5^ and ∼4 × 10^−4^ respectively (Bebenek et al., 1990; Lee et al., 2016), but with the polymerase fidelity determination dependent upon the assay (Lee et al., 2016). Here the median error rates seen for 1 G+ and 1 G- were 2.1 × 10^−5^ and 3.8 × 10^−5^ respectively. Furthermore, we suggest that reaction conditions could influence the baseline error rate. As such, various protocols for determining fidelity such as the Kunkel method may be prone to over- or underrepresent the effects that specific substitution errors have on fidelity. Regardless, the concomitant internal consistency in the fidelity determination methodology used for this study and the dependable differences in error rate between gravitational conditions further strengthens our conclusions.

Parsing of the DNA polymerization data by specific substitution error identity gave further insight into the makeup of the differences in polymerase fidelity in 1 G *vs.* μG conditions. Of 12 possible substitutions, μG- samples showed reduced fidelity due to microgravity in 9, generally corresponding to substitutions with A, C or G in place of the templated base, compared to 1 G (either + or -) samples (Figure 4; all statistics not shown). In addition, for 7 substitutions in μG+ samples, polymerization fidelity was decreased compared to μG-. The overall error rate of each sample, however, depended more on high frequency substitutions (*i.e.* C → A), rather than low-frequency mutations (*i.e*. A → C). The sectioning of error rates by substitution lent further credence to the assertion that less accurate polymerization in microgravity is only partially masked in enzymes bearing the exonuclease domain.

The directionality of mean error rate for each substitution as a result of polymerization with both Klenow (exo + and -) enzymes follows a general trend of decreased fidelity in substitutions resulting in insertion of A, C or G in place of a templated base. Yet, insertion of T in place of all possible template bases occurred (often non-significantly) at a decreased rate, or alternatively, was increased by a relatively small margin in microgravity. This corresponds to an increased propensity for polymerases to incorrectly incorporate dTTP, dGTP, and dCTP in microgravity, while being more sensitive to dATP (corresponding to a T insertion on the template strand in subsequent rounds of polymerization). Coupled with previously discussed results, this suggests that whether by polymerization of templated A residues, or by insertion of dATPs, DNA polymerization in microgravity proceeds with increased accuracy when involving this base, by an unknown mechanism. This is seemingly contradictory to the “A-rule” of DNA polymerization and *trans*-lesion synthesis which has been observed under earth-like gravity, whereby dATP is preferentially incorporated on template lesions (Sheriff et al., 2008). As such, it may be the case that this rule is not applicable in microgravity, and further prompts structural investigation of the Klenow fragment in microgravity in comparison to 1 G to resolve putative differences in binary complex structures of this enzyme with each of the incoming dNTPs between gravitational states.

**FIGURE 7:**
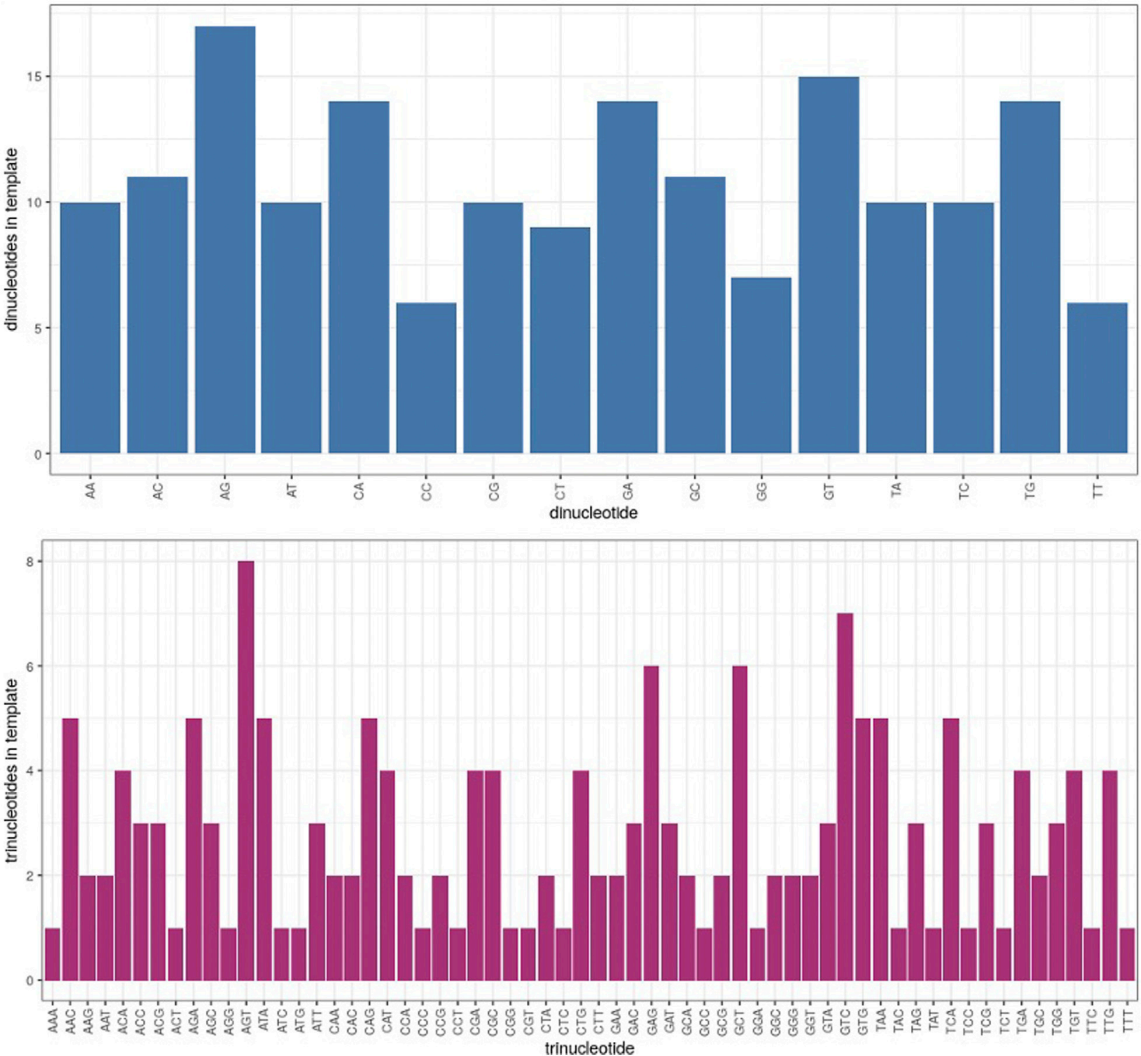
Distribution of dinucleotides (blue) and trinucleotides (magenta) in template strand, listed in alphabetical order according to the nucleotide pairs (*ie.* AA, AC, AG etc. and AAA, AAC, AAG etc.). The *Y* axis represents the number of times a given di- or trinucleotide occurred in the 175 bp template region which was analyzed.

Because the context of nucleotide sequences may have an influence on differential fidelity in microgravity, computation of di- and trinucleotide error rates in each test sample was a further useful analytical tool. Of 16 possible dinucleotide combinations, 12 were significantly more error prone in μG- than 1 G- samples, while only 7 dinucleotides exhibited an increased error rate in μG + compared to 1 G+ (Figure 5B). Similarly, of the 64 possible trinucleotide sequences, 30 were significantly more error-prone under μG- conditions, while 13 were so in μG +vs. 1G+ (not shown). Both μG+ and μG- samples exhibited significantly less accuracy for both di- and trinucleotides compared to their 1 G counterparts. The magnitude of the significance values also decreased from mono- → di- → trinucleotide, indicating that a potentially more faithful error rate determination was achieved as nucleotide length was increased. We acknowledge, however, that di- and trinucleotide distributions in the template were uneven, and considerations of this for future template design may be warranted (Figure 7). Nonetheless, this evidence underscores the importance of di- and trinucleotide error rate determinations for investigations of polymerase error rates in space. As well, the context and frequency of specific nucleotide sequences may also be reflected in the error scanning across the 175 bp template (Figure 3A), with μG + tracking above 1 G+, and μG- above 1 G-, underscoring again the detrimental effect of microgravity and the capacity of proofreading capacity to only partially “rescue” this effect.

Analysis of deletion mutations revealed that both μG+ and μG- samples were significantly more prone to deletions than their 1 G counterparts (Figure 6). When separation of deletion error rate by base identity was conducted, a substantial difference in error rate was identified between purine and pyrimidine bases with both polymerases. While deletion of purines was not significantly increased in μG+, this test case resulted in significantly more pyrimidine mutations in microgravity than in 1 G. Overall, this effect was less obvious as purine deletions occurred at a substantially higher frequency than for pyrimidines. With regards to Klenow (exo-), all possible base deletions were more frequent in microgravity. Deletion mutations are generally thought to be introduced by strand slippage during DNA polymerization, resulting in a single base “flipped out” of the double helix so as to generate an extension from the next base in the template, possibly precipitated by specific interactions with dNTPs (Garcia-Diaz et al., 2006; Bebenek et al., 2008). We speculate that DNA polymerization in microgravity could facilitate C and T purine flipping and subsequent template slippage, resulting in an increased deletion rate.

Error analysis argues that microgravity induces alterations in DNA polymerase fidelity that were only partially alleviated by the proofreading 3′→5′ exonuclease domain. However, it is unknown whether this effect would be observable in other polymerases from prokarya and eukarya. It is possible that the *E. coli* DNA polymerase, as a family A polymerase, may share this effect with DNA polymerase I enzymes from other bacterial species, DNA T7 polymerase and eukaryotic Pol γ, θ, and *v*. Yet, given the prokaryotic origin of mitochondria, in addition to numerous studies highlighting increased mitochondrial ROS production in microgravity, the fidelity of polymerases associated with this organelle in space may be of specific concern (Mao et al., 2013). Interestingly, Pol γ is essential for mtDNA replication, and dysfunction of this enzyme is the cause of numerous autosomal mitochondrial diseases (Zhang et al., 2011). Furthermore, the expression of the *POLG* gene which encodes this polymerase has been shown to be downregulated in human T cells during SMG culture (Ward et al., 2006). Because Pol γ possesses a 3`→5′ exonuclease domain and not 5`→3′ exonuclease activity, it may perform more poorly in a template-dependent manner, and in specific trinucleotide contexts, as discussed previously in the context of Klenow (exo+), which possesses the same functionality as Pol γ. As such, given the essential nature of this polymerase, its similarities in domain architecture to Klenow (exo+) used in this experiment, its putative involvement in the microgravity phenotype, and the oxidative risk to mtDNA in space, the results of this study may have direct applicability here, and may partially explain previous mitochondrial dysfunction in microgravity more broadly.

**FIGURE 8:**
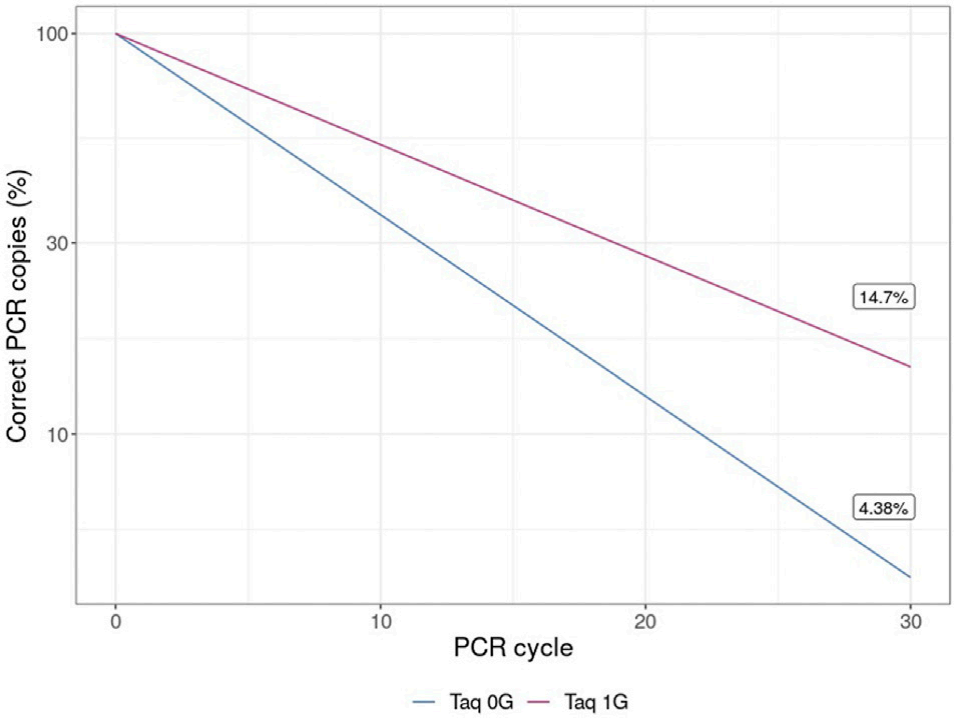
Percentage of templates containing errors over 30 cycles of PCR with *Taq* polymerase. The theoretical decreased fidelity due to PCR was calculated based on mean increase in error rate from either earth-like gravity (1 G) or microgravity (μG) and adjusted based on the documented error rate for *Taq* polymerase.

The results of this experiment also have applicability in biotechnology. *Taq* polymerase, the most commonly used enzyme for conducting PCR, is a 3`→5′ proofreading exonuclease-deficient DNA polymerase I derived from *Thermus aquaticus.* Therefore, the increase in the error rate of Klenow (exo-) in microgravity observed here may be applicable to the fidelity of *Taq* polymerase-mediated PCR in microgravity and is therefore relevant for future biological research in space. Even the relatively small decrease in polymerase fidelity observed in microgravity of Klenow (exo-), when adjusted based on the fidelity of *Taq* polymerase and propagated over 30 cycles for a 1 Kb template, yields a concerning ∼335% increase in incorrect templates in solution (Figure 8).

DNA polymerase I also shares functional characteristics with eukaryotic Pol β, as both act as key repair-oriented polymerases, and also act to resolve Okazaki fragments during replication. Many polymerases are highly evolutionarily conserved, but further investigation of whether both replicative and repair polymerases from all families display decreased polymerization fidelities in microgravity is warranted. It is also unknown if the 1.1 and 2.4-fold increases in respective substitution error rates of Klenow (exo+) and (exo-) seen here would pose a significant risk to organisms including astronauts. However, if the effect is broadly applicable to other polymerases, the differential in error rate could be higher. Furthermore, a far greater risk of reduced polymerization fidelity would be in the generation of deletion errors since this results in frameshift mutations in protein-coding genes. Polymerases such as Pol δ have evolved to tolerate strand slippage during repair of DSBs, and thus introduce a high rate of deletion errors (Garcia-Diaz et al., 2006). Given the increased radiation risk in space, if repair of critical DSBs introduces more deletion errors in microgravity than on earth, that effect alone could have drastic consequences to astronaut health on extended periods in space such as future missions to the Moon and Mars.

## Data Availability

The data presented in the study are deposited in the Sequence Read Archive (SRA), accession number: prjna773952.

## References

[B1] Battista N. Meloni M. A. Bari M. Mastrangelo N. Galleri G. Rapino C. (2012). 5‐Lipoxygenase‐dependent Apoptosis of Human Lymphocytes in the International Space Station: Data from the ROALD experiment. FASEB j. 26 (5), 1791–1798. 10.1096/fj.11-199406 22253478

[B2] Bebenek K. Garcia‐Diaz M. Foley M. C. Pedersen L. C. Schlick T. Kunkel T. A. (2008). Substrate‐induced DNA Strand Misalignment during Catalytic Cycling by DNA Polymerase λ. EMBO Rep. 9 (5), 459–464. 10.1038/embor.2008.33 18369368 PMC2278112

[B3] Bebenek K. Joyce C. M. Fitzgerald M. P. Kunkel T. A. (1990). The Fidelity of DNA Synthesis Catalyzed by Derivatives of *Escherichia coli* DNA Polymerase I. J. Biol. Chem. 265 (23), 13878–13887. 10.1016/s0021-9258(18)77430-9 2199444

[B4] Cannan W. J. Pederson D. S. (2016). Mechanisms and Consequences of Double-Strand DNA Break Formation in Chromatin. J. Cell. Physiol. 231 (1), 3–14. 10.1002/jcp.25048.Mechanisms 26040249 PMC4994891

[B5] Chan P. P. Holmes A. D. Smith A. M. Tran D. Lowe T. M. (2012). The UCSC Archaeal Genome Browser: 2012 Update. Nucleic Acids Res. 40 (D1), D646–D652. 10.1093/nar/gkr990 22080555 PMC3245099

[B6] Costello M. Pugh T. J. Fennell T. J. Stewart C. Lichtenstein L. Meldrim J. C. (2013). Discovery and Characterization of Artifactual Mutations in Deep Coverage Targeted Capture Sequencing Data Due to Oxidative DNA Damage during Sample Preparation. Nucleic Acids Res. 41 (6), e67. 10.1093/nar/gks1443 23303777 PMC3616734

[B7] Dahlberg M. E. Benkovic S. J. (1991). Kinetic Mechanism of DNA Polymerase I(Klenow Fragment): Identification of a Second Conformational Change and Evaluation of the Internal Equilibrium Constant. Biochemistry 30 (20), 4835–4843. 10.1021/bi00234a002 1645180

[B8] Derbyshire V. Freemont P. S. Sanderson M. R. Beese L. Friedman J. M. Joyce C. M. (1988). Genetic and Crystallographic Studies of the 3′,5′-Exonucleolytic Site of DNA Polymerase I. Science 240 (4849), 199–201. 10.1126/science.2832946 2832946

[B9] Dextraze M.-E. Gantchev T. Girouard S. Hunting D. (2010). DNA Interstrand Cross-Links Induced by Ionizing Radiation: An Unsung Lesion. Mutat. Research/Reviews Mutat. Res. 704 (1–3), 101–107. 10.1016/j.mrrev.2009.12.007 20079875

[B10] Eckert K. A. Kunkel T. A. (1991). DNA Polymerase Fidelity and the Polymerase Chain Reaction. Genome Res. 1 (1), 17–24. 10.1101/gr.1.1.17 1842916

[B11] Garcia-Diaz M. Bebenek K. Krahn J. M. Pedersen L. C. Kunkel T. A. (2006). Structural Analysis of Strand Misalignment during DNA Synthesis by a Human DNA Polymerase. Cell 124 (2), 331–342. 10.1016/j.cell.2005.10.039 16439207

[B12] Garcia-Diaz M. Bebenek K. (2007). Multiple Functions of DNA Polymerases. Crit. Rev. Plant Sci. 26 (2), 105–122. 10.1080/07352680701252817 18496613 PMC2391090

[B13] Horneck G. Rettberg P. Kozubek S. Baumstark-Khan C. Rink H. Schäfer M. (1997). The Influence of Microgravity on Repair of Radiation-Induced DNA Damage in Bacteria and Human Fibroblasts. Radiat. Res. 147 (3), 376–384. 10.2307/3579347 9052686

[B14] Kennedy A. R. (2014). Biological Effects of Space Radiation and Development of Effective Countermeasures. Life Sci. Space Res. 1 (1), 10–43. 10.1016/j.lssr.2014.02.004 PMC4170231 25258703

[B15] Langmead B. Salzberg S. L. (2012). Fast Gapped-Read Alignment with Bowtie 2. Nat. Methods 9 (4), 357–359. 10.1038/nmeth.1923 22388286 PMC3322381

[B16] Lee D. F. Lu J. Chang S. Loparo J. J. Xie X. S. (2016). Mapping DNA Polymerase Errors by Single-Molecule Sequencing. Nucleic Acids Res. 44 (13), e118. 10.1093/nar/gkw436 27185891 PMC5291262

[B17] Li N. An L. Hang H. (2015). Increased Sensitivity of DNA Damage Response-Deficient Cells to Stimulated Microgravity-Induced DNA Lesions. PLoS ONE 10 (4), e0125236. 10.1371/journal.pone.0125236 25915950 PMC4411073

[B18] Li Z. Jella K. K. Jaafar L. Li S. Park S. Story M. D. (2018). Exposure to Galactic Cosmic Radiation Compromises DNA Repair and Increases the Potential for Oncogenic Chromosomal Rearrangement in Bronchial Epithelial Cells. Sci. Rep. 8, 11038. 10.1038/s41598-018-29350-5 30038404 PMC6056477

[B19] Mao X. W. Pecaut M. J. Stodieck L. S. Ferguson V. L. Bateman T. A. Bouxsein M. (2013). Spaceflight Environment Induces Mitochondrial Oxidative Damage in Ocular Tissue. Radiat. Res. 180 (4), 340–350. 10.1667/RR3309.1 24033191

[B20] Moreno-Villanueva M. Wong M. Lu T. Zhang Y. Wu H. (2017). Interplay of Space Radiation and Microgravity in DNA Damage and DNA Damage Response. Npj Microgravity 3 (14). 10.1038/s41526-017-0019-7 PMC5460239 28649636

[B21] Ohnishi T. Takahashi A. Nagamatsu A. Omori K. Suzuki H. Shimazu T. (2009). Detection of Space Radiation-Induced Double Strand Breaks as a Track in Cell Nucleus. Biochem. Biophysical Res. Commun. 390 (3), 485–488. 10.1016/j.bbrc.2009.09.114 19799866

[B22] Ohnishi T. Takahashi A. Ohnishi K. Takahashi S. Masukawa M. Sekikawa K. (2001). Alkylating Agent (MNU)-induced Mutation in Space Environment. Adv. Space Res. 28 (4), 563–568. 10.1016/S0273-1177(01)00392-1 11799989

[B23] Potapov V. Ong J. L. (2017). Examining Sources of Error in PCR by Single-Molecule Sequencing. PLoS ONE 12 (1), e0169774. 10.1371/journal.pone.0169774 28060945 PMC5218489

[B24] Pouget J.-P. Frelon S. Ravanat J.-L. Testard I. Odin F. Cadet J. (2002). Formation of Modified DNA Bases in Cells Exposed Either to Gamma Radiation or to High-LET Particles1. Radiat. Res. 157 (5), 589–595. 10.1667/0033-7587(2002)157[0589:fomdbi]2.0.co;2 11966325

[B25] Pray L. A. (2008). DNA Replication and Causes of Mutation. Nat. Education 1 (1), 214.

[B26] Pross H. D. Casares A. Kiefer J. (2000). Induction and Repair of DNA Double-Strand Breaks under Irradiation and Microgravity. Radiat. Res. 153, 521–525. 10.1667/0033-7587(2000)153[0521:iarodd]2.0.co;2 10790272

[B27] SantaLucia J. Jr (1998). A Unified View of Polymer, Dumbbell, and Oligonucleotide DNA Nearest-Neighbor Thermodynamics. Proc. Natl. Acad. Sci. 95 (4), 1460–1465. 10.1073/pnas.95.4.1460 9465037 PMC19045

[B28] Santivasi W. L. Xia F. (2014). Ionizing Radiation-Induced DNA Damage, Response, and Repair. Antioxid. Redox Signaling 21 (2), 251–259. 10.1089/ars.2013.5668 24180216

[B29] Sheriff A. Motea E. Lee I. Berdis A. J. (2008). Mechanism and Dynamics of Translesion DNA Synthesis Catalyzed by the *Escherichia coli* Klenow Fragment. Biochemistry 47 (33), 8527–8537. 10.1021/bi800324r 18652487 PMC2692461

[B30] Singh K. Srivastava A. Patel S. S. Modak M. J. (2007). Participation of the Fingers Subdomain of *Escherichia coli* DNA Polymerase I in the Strand Displacement Synthesis of DNA. J. Biol. Chem. 282 (14), 10594–10604. 10.1074/jbc.M611242200 17259182

[B31] Takahashi A. Ikeda H. Yoshida Y. (2018). Role of High-Linear Energy Transfer Radiobiology in Space Radiation Exposure Risks. Int. J. Part. Ther. 5 (1), 151–159. 10.14338/IJPT-18-00013.1 31773027 PMC6871591

[B32] Todd P. (1989). Gravity-dependent Phenomena at the Scale of the Single Cell. Am. Soc. Gravit. Space Biol. Bull. 2, 95–113. 10.1109/ISBI.2011.5872826 11540086

[B33] Ward N. E. Pellis N. R. Risin S. A. Risin D. (2006). Gene Expression Alterations in Activated Human T-Cells Induced by Modeled Microgravity. J. Cell. Biochem. 99 (4), 1187–1202. 10.1002/jcb.20988 16795038

[B34] Zhang L. Chan S. S. L. Wolff D. J. (2011). Mitochondrial Disorders of DNA Polymerase γ Dysfunction: From Anatomic to Molecular Pathology Diagnosis. Arch. Pathol. 135 (7), 925–934. 10.5858/2010-0356-rar.1 PMC3158670 21732785

