# Supplementary material for: Fidelity of a Bacterial DNA Polymerase in Microgravity, a Model for Human Health in Space
Source: Front Cell Dev Biol. 2021 Nov 29;9:702849. doi: 10.3389/fcell.2021.702849 (PMC8666419; doi:10.3389/fcell.2021.702849)
Supplement: Supplementary file 1 [file DataSheet2.PDF]

Supplementary Methods:

UMI-barcoded ssDNA Template Library Design and Generation:

Amplification of the PolERIS Template for Further Testing:

The PolERIS template (synthesized by Synbio Technologies Inc., Monmouth Junction, NJ, USA) cloned into a pUC-57 vector and was subsequently sequence verified. Amplification was completed by polymerase chain reaction (PCR) using Q_5 DNA Polymerase Hot-Start Mastermix (New England Biolabs, Ipswitch, MA, USA) with 500 nM each of M13 forward primer (TGTAAAACGACGGCCAGT) and pre-AMP-R reverse primer (GCTGAAAGTAAGGCAGCC; both from Integrated DNA Technologies, Coralville, IA, USA), with 1 ng (0.5 fmol) of template-containing plasmid in a 25 μL reaction. Cycling conditions were as follows: initial denaturation for 1 min at 98 ˚C, 25 cycles of denaturation for 10 sec at 98 ˚C, annealing for 20 sec at 64 ˚C, and extension for 30 sec at 72 ˚C, followed by a final extension at 72 ˚C for 2 min. The amplified product was purified using solid phase reverse-immobilization (SPRI) magnetic beads (GE Healthcare, Mississauga, ON, Canada), and referred to as 'PolERIS pre-DNA'.

Assembly of the UMI-barcoded Polymerization Template:

A secondary PCR amplification was completed to append a random 20 bp DNA unique molecular identifier (UMI) and polymerization start site to the template. The PolERIS pre-DNA was PCR-amplified using NEBNext Ultra II Q_5 DNA Polymerase Hot-Start Mastermix (New England Biolabs). PCR was completed in a 200 μL reaction volume with 200 nM of FWD-I primer (AGTGGTTACGGTCAGCAGTTCGGG) and RVS 4.0 primer

(pGCGTTAGTTAAAGGGCCTCACTGTCGATGGCATAGATCCAGCGGAGTTCAATCGCTTCGGCAACGNNNNNNNNNNNNNNNNNNNNAAGGCAGCCAGTATCTTGACC; both Integrated DNA Technologies), as well as 50 pg (~0.77 amol) of template DNA. The FWD-I primer had 6 consecutive phosphorothioate linkages at its 5′ end to hinder exonucleolytic degradation. Cycling conditions were as follows: initial denaturation for 1 min at 98 ˚C, 20 cycles of denaturation for 10 sec at 98 ˚C and annealing/extension for 50 sec at 69 ˚C, followed by a final extension at 69 ˚C for 2 min. The amplified product was purified using SPRI magnetic beads and was denoted as PolERIS dsDNA-1, with the concentration quantified using the dsDNA HS assay (Invitrogen, Carlsbad, CA, USA) on a Qubit 3.0 fluorimeter (Life Technologies, Carlsbad, CA, USA).

Amplification of the PolERIS Polymerization Template:

A third PCR was completed using 200 nM of FWD-I primer and RVS-P primer (pCTGTCGATGGCATAGATCCAGC; Biobasic Inc., Markham, ON, Canada). The reverse primer was purified with high-performance liquid chromatography (HPLC) and assessed for purity using analytical capillary electrophoresis at Biobasic Inc. PCR was conducted in a 1 mL reaction volume using NEBNext Ultra II Q_5 DNA Polymerase Hot-Start Mastermix. To faithfully conserve the diversity of 20 bp UMIs in the PolERIS dsDNA-1 library, 1.2 µg (2.1 pmol) of template was used to represent the approximately $4^{20} \approx 1.1$ trillion unique sequences in solution. Cycling conditions were as follows: initial denaturation for 1 min at 98 ˚C, 8 cycles of denaturation for 10 sec at 98 ˚C and annealing/extension for 50 sec at 69 ˚C, followed by a final extension at 69 ˚C for 2 min. The product was purified using SPRI magnetic beads and denoted

as PolERIS dsDNA-2. As well, dsDNA concentration was quantified using the dsDNA HS assay on a Qubit 3.0 fluorimeter, as described.

<u>Nucleolytic Digestion to Yield Pure ssDNA:</u>

In order to generate ssDNA from the PolERIS dsDNA-2 template library, 20 µg (~36 pmol) of template was digested with 100 U of lambda exonuclease (New England Biolabs) in a 500 µL reaction for 2 h at 37 ˚C. Buffer conditions were as follows: 67 mM glycine-KOH (pH 9.4), 2.5 mM $MgCl_2$ and 0.01% (v/v) Triton X-100. Reactions were then adjusted to pH ~7.5 by addition of 3 µL 1N HCl. l.5 µL of dsDNAse (unit definition not provided by supplier, ThermoFisher Scientific), a double-stranded DNA-specific nuclease, was then added to the reaction and mixed at 37 ˚C to further degrade residual dsDNA molecules, yielding a highly pure ssDNA template library. The reaction incubated for 10 min at 37 ˚C and then heat-inactivated at 65 ˚C for 25 min in order to halt all nucleolytic activity in the reaction mixture. The reaction was purified using SPRI magnetic beads. As well, ssDNA concentration was quantified using the ssDNA assay on a Qubit 3.0 fluorimeter. The final assembled ssDNA sequence (with UMI and primer binding site) is shown in Supplementary Figure 1.

Sample Post-Flight Processing:

<u>Exonuclease VII Digestion:</u>

Samples were digested with exonuclease VII (New England Biolabs) to trim both 5′ and 3′ single-stranded tails. To each 25 µL reaction, 2 U of exonuclease VII were added and incubated at 37 ˚C for one hour to ensure complete digestion. Exonuclease VII was chosen due to its

bidirectional behavior, which leaves small (≤10 nt) overhangs on its products. Therefore, following digestion, all reaction products were trimmed on the basis of nascent strand length. Samples were then purified using 1X SPRI magnetic beads in 16.7 μL volumes.

End-Repair and Adaptor Ligation for Illumina Sequencing:

Samples were processed using the standard protocol for end-repair and adaptor ligation stipulated for the NEBNext Ultra II DNA Library preparation kit. Standard NEBNext adaptors were used, which were included in the NEBNext Multiplex Oligos (Dual Index Primer Set 1) package (New England Biolabs). Following DNA end-preparation and adaptor ligation, samples were again purified with SPRI magnetic beads. All libraries were prepared in triplicate.

Library Quantitation, Dilution and Amplification:

Library quantitation and subsequent dilution of each sample was imperative to ensure that each replicated DNA molecule was copied to yield consensus sequencing reads with sufficiently large sample sizes to limit sequencing errors to a negligible amount. Conversely, solutions containing sub-optimal amounts of DNA molecules were also not desirable, as this could limit error-rate determination accuracy for high-fidelity polymerases. In order to minimize and detect variability in this process, each adaptor-ligated sample was amplified in triplicate. The NEBNext Library Quantitation Kit (New England Biolabs) was utilized to accurately quantify all libraries by qPCR, independent of library insert sizes. Using the DNA library standards included in the kit, each library was quantified using a CFX 96 qPCR thermocycler (BioRad, Hercules, CA, USA). The concentration of each library was calculated and then diluted so that 7.5 μL volumes contained 4×10^8 bp. These were then used as templates in into individual PCR reactions. In turn these used NEBNext Ultra II Q_5 DNA Polymerase Hot-Start Mastermix in 25 μL volumes. Each

sample was amplified with using unique primer pairs included in the NEBNext Multiplex Oligos package. Amplified products were again purified using SPRI magnetic beads, and quantified using the dsDNA HS assay on a Qubit 3.0 fluorimeter.

Sequencing:

Following library purification, samples were pooled in equal concentrations at a final molarity of 40 nM and Illumina analysis was conducted at the Center for Applied Genomics (TCAG, SickKids Hospital, Toronto, Canada). The NovaSeq™ 6000 sequencer (Illumina, San Diego, CA, USA), with SP flow cell was used for paired-end sequencing.
