# Supplementary material for: Fidelity of a Bacterial DNA Polymerase in Microgravity, a Model for Human Health in Space
Source: Front Cell Dev Biol. 2021 Nov 29;9:702849. doi: 10.3389/fcell.2021.702849 (PMC8666419; doi:10.3389/fcell.2021.702849)
Supplement: Supplementary file 2 [file DataSheet3.pdf]

5′-AGTGGTTACGGTCAGCAGTTCGGGCTTAGTTGTCGGTGCTATTGCCGTGTCCTCGTGAGTGGTAGA
GTTGCGTGTTGGAAGTAGGGAGGGTTCCTGGTCATAGAAGAGAGGGCATCCAGAAACAGAGTCGGGTAA
GTGTCACATCAAATAGGGAGTAGAGTGTAGTCGGAAAGCCAATCGGTCATCATCGGACGCACAGCGGAA
CAACACGAACTGCTTCCTCTCATTACGAGATTTACCACTGTATTCCCATCGGCAACTGCTCAGGCTGCT
CCCTCAGACTGTAGATACGCTAATCATACTTGTCGTGCTTGTAACCCGCTCGGTTCGCTAACTTGCTCC
ATCTTAGTGGGATCAGCTTATCGTGGTTTATGGTCTCATGCCTGTTGTCTGTTGGTCAAGGTGTTCGAG
GTAGTGGCCTCCTAGTTCCGATCTCTCTGAGGGCGTCATCTCAATTACGACTTAACGAGGAATCTCCGT
ATCTGGAATAAGCCTCCATCACTTCACATCTAACAACTCTCACCTCTGGTGGCACTTACACGTCATGTA
CGACCGAGCCAGGCGGTAGGAGTACCCAGGACGCTTCTCGCTTTGAGCCCATAACCATCAGACGACCTG
AGATATAGATGAGAGTCAGTAACACCGAGCAGTCGCTGCGCTAGTCAACACGAGTGAATAACAGTCCGA
ACAGTGTGTAGTGGCATTGTGCTAAAGCGTCGGGGTCATTGTAATTGGTCAAGATACTGGCTGCCTT
NNNNNNNNNNNNNNNNNNNN**CGTTGCCGAAGCGATTGAACTCC**GCTGGATCTATGCCATCGACAG -3′

**Supplementary Figure 1:** QRGX ssDNA template. The template strand (white background) was placed upstream of the UMI (light grey) and the polymerization primer binding site (bold). The QRGX RVS-P primer binding site was also present as a residual product of ssDNA assembly (underlined).

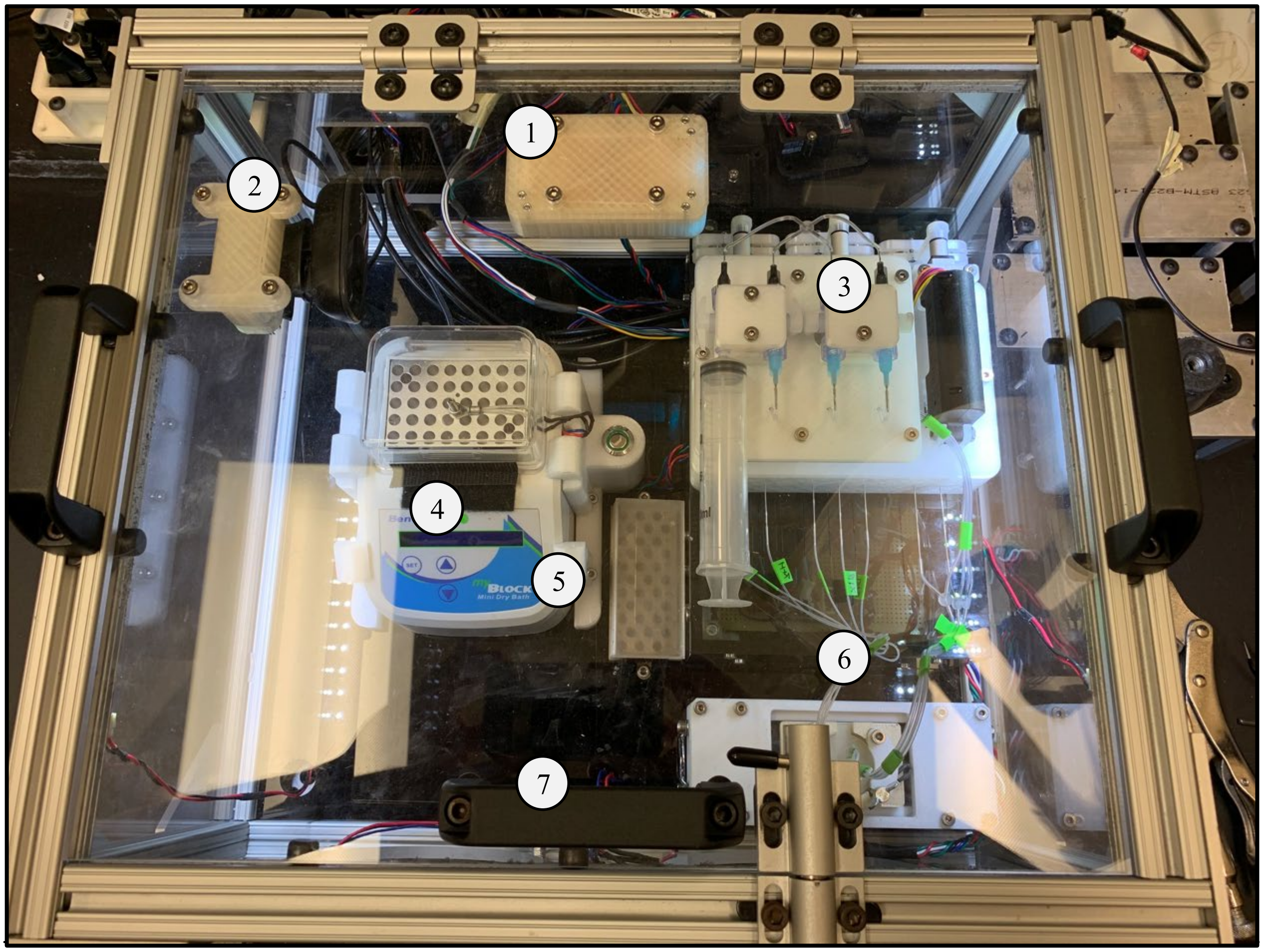


**Supplementary Figure 2:** Photograph of the payload designed to assess the fidelity of DNA polymerase in microgravity. 1: Onboard telemetry display. 2: Webcam 3: Injector system and motherboard (not shown, below injector). 4: Dry-block heater. 5: Sample storage. 6: Thermal containment unit.

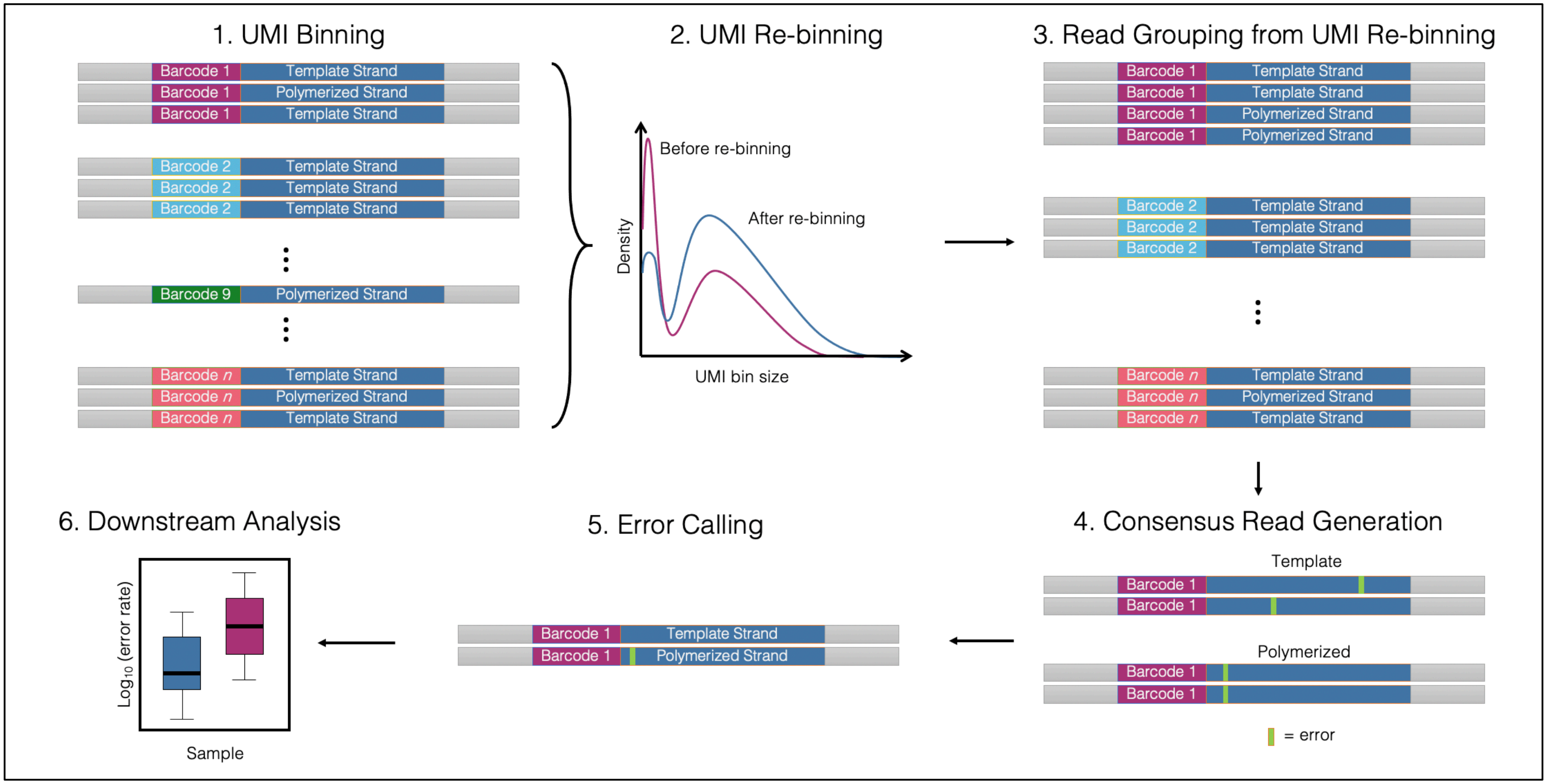


**Supplementary Figure 3:** UMI binning, re-binning, consensus read generation, and error calling scheme. All reads were binned based on UMI identity (1), and the frequency of each UMI was recorded. Singleton UMI bins which were within one base mismatch of exactly one larger UMI bin were re-binned, and the distribution of UMI frequencies was compared before and after re-binning (2). UMIs were then grouped based on their resulting designations after re-binning (3). UMI bins without adequate numbers of both template and polymerized strands were discarded, and consensus reads for remaining bins were generated (4). Errors were then called in the event of a substitution, insertion, or deletion between the template and polymerized strand (5). Tabulation of the nature of each error, its locus on the template, and statistical information were utilized for downstream analyses (6).

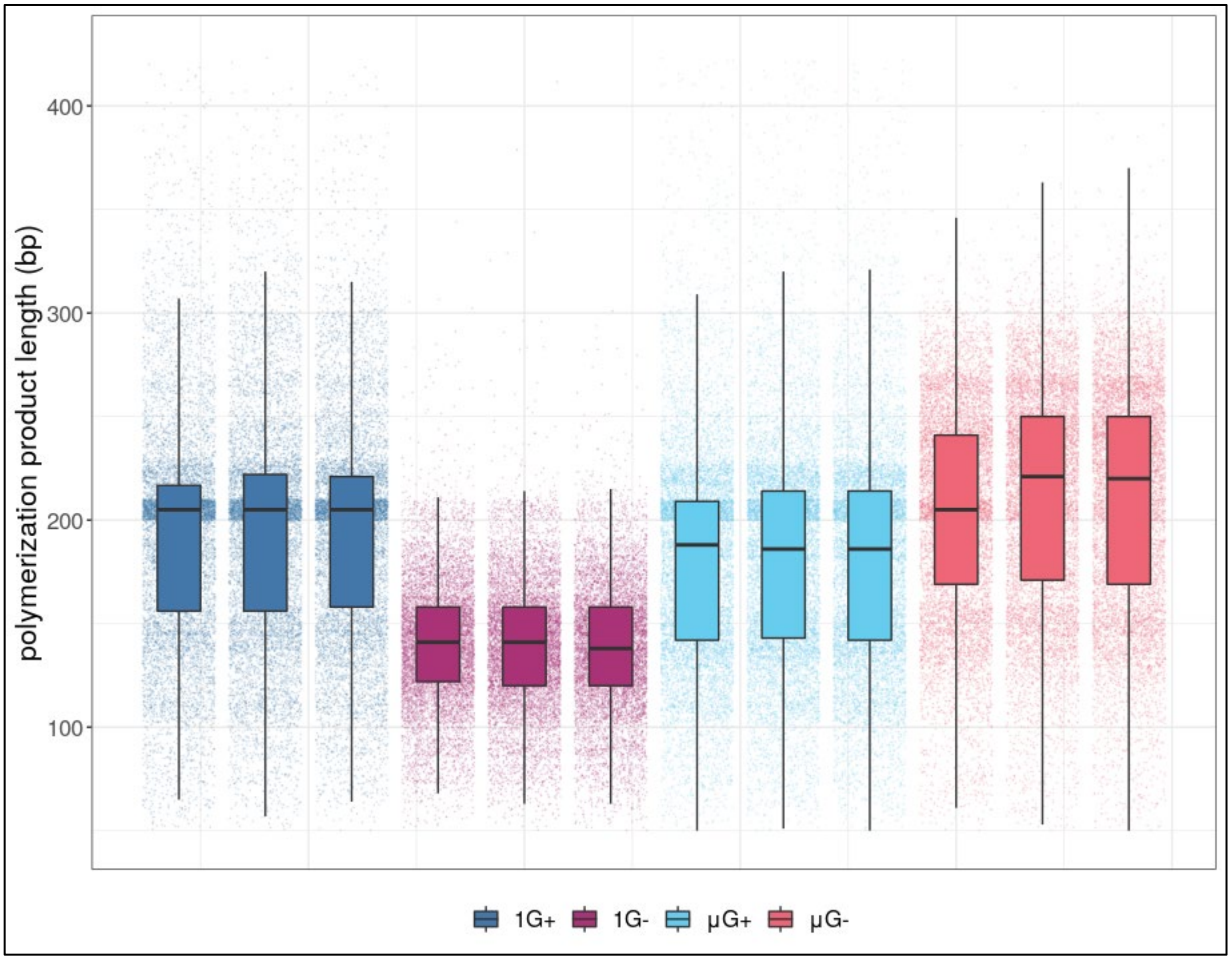


**Supplementary Figure 4:** Boxplots of polymerization product length distributions for each test condition. Samples are labeled μG or 1G, with + and – referring to the presence or absence of exonuclease activity in the polymerase, respectively. Superimposed jitter plots are representative of individual template lengths in each of the triplicate sequencing libraries which were prepared for each test condition.

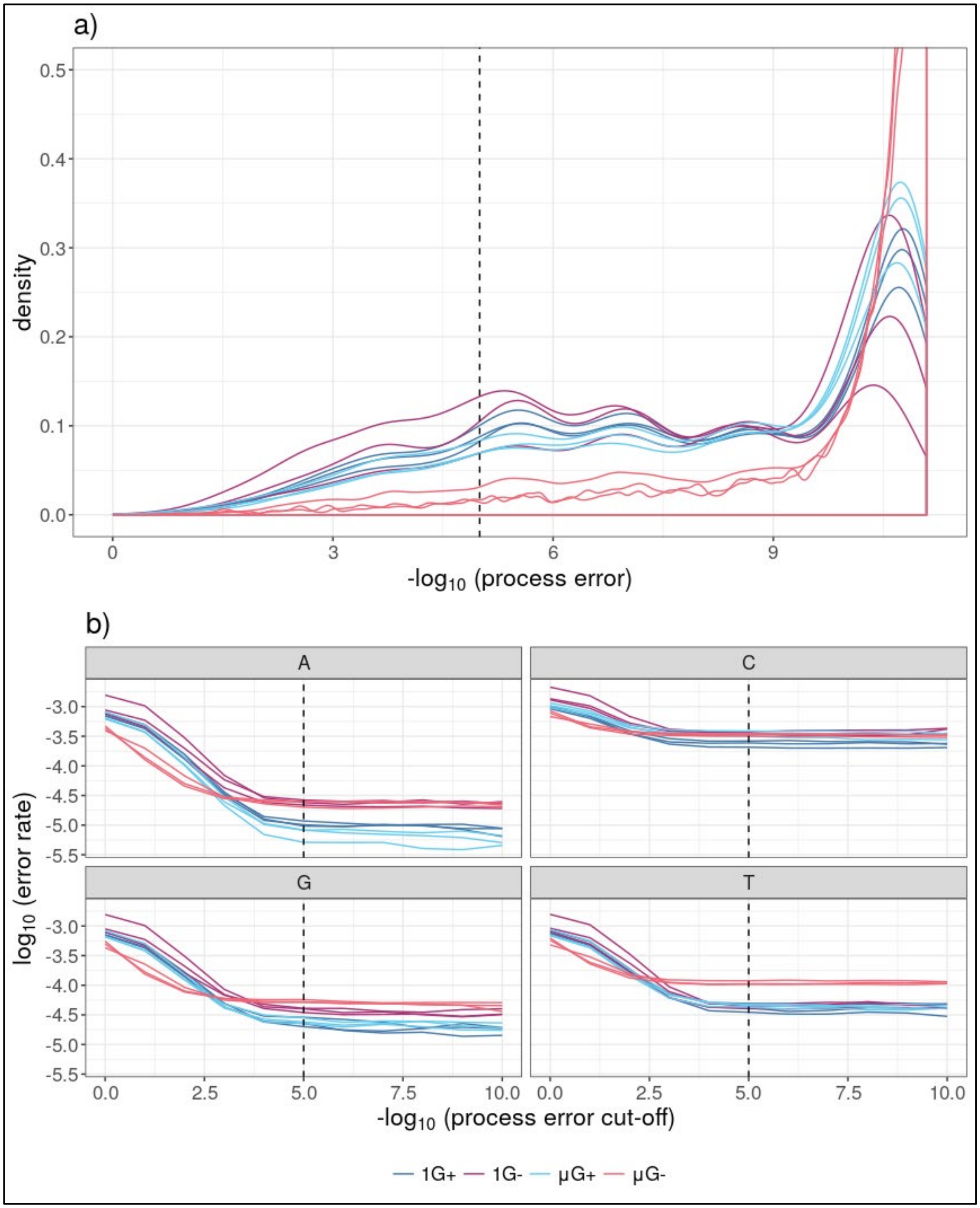


**Supplementary Figure 5:** Expected error rates in each sample (μG or 1G with + and – referring to the presence or absence of exonuclease activity in the polymerase, respectively). **a.** Expected error rates given $P_E$ thresholds in each triplicate library for each sample. The chosen $P_E$ cut-off of $1 \cdot 10^{-4}$ is shown as a dotted black line. **b.** Density plots of expected error for each sample with respect to each nucleotide.
