# Supplementary material for: Fidelity of a Bacterial DNA Polymerase in Microgravity, a Model for Human Health in Space
Source: Front Cell Dev Biol. 2021 Nov 29;9:702849. doi: 10.3389/fcell.2021.702849 (PMC8666419; doi:10.3389/fcell.2021.702849)
Supplement: Supplementary file 3 [file DataSheet1.PDF]

Supplementary Data

QRGXbowtie2aligner.sh

This script is ran in a folder containing a subdirectory with all paired-end .fastq.gz files in it, labelled QRGX1 through 12.

```
#!/bin/bash
# QRGXbowtie2aligner
cd SAM\ files //change to destination directory

for j in {1..12..3}
do
  for ((i=j;i<j+3;i++))
  do
  bowtie2 --local -x /home/rstudio/reference_data/QRGX -1 <(zcat "raw_data/QRGX${i}_R1.fastq.gz") -2 <(zcat "raw_data/QRGX${i}_R2.fastq.gz") --xeq -p 7 --very-sensitive | sed '3 a @1\t2\t3\t4\t5\t6\t7\t8\t9\t10\t11\t12\t13\t14\t15\t16\t17\t18\t19\t20\t21\t22\t23' | gzip > "QRGX${i}.sam.gz" &
  done
  wait
done
```

QRGXAnalyzeAll.sh

Following alignment using QRGXbowtie2aligner.sh, this script is ran to call errors in each of the resulting .bam files.

```
#!/bin/bash
# QRGXAnalyzeAll
 for ((i=1;i<=12;i++))
 do
 Rscript ErrorRateCaller.R $i 7 &
 done
```

QRGX_reference.fasta

This is a copy of the .fasta file containing the QRGX reference sequence with degenerate UMI and polymerization primer binding site attached

```
>QRGX_reference

AGTGGTTACGGTCAGCAGTTCGGGCTTAGTTGTCGGTGCTATTGCCGTGTCCTCGTGAGTGGTAGAGTTGCGTGTTG
GAAGTAGGGAGGGTTCCTGGTCATAGAAGAGAGGGCATCCAGAAACAGAGTCGGGTAAGTGTCACATCAAATAGGGA
GTAGAGTGTAGTCGGAAAGCCAATCGGTCATCATCGGACGCACAGCGGAACAACACGAACTGCTTCCTCTCATTACG
AGATTTACCACTGTATTCCCATCGGCAACTGCTCAGGCTGCTCCCTCAGACTGTAGATACGCTAATCATACTTGTCG
TGCTTGTAACCCGCTCGGTTCGCTAACTTGCTCCATCTTAGTGGGATCAGCTTATCGTGGTTTATGGTCTCATGCCT
GTTGTCTGTTGGTCAAGGTGTTCGAGGTAGTGGCCTCCTAGTTCCGATCTCTCTGAGGGCGTCATCTCAATTACGAC
TTAACGAGGAATCTCCGTATCTGGAATAAGCCTCCATCACTTCACATCTAACAACTCTCACCTCTGGTGGCACTTAC
ACGTCATGTACGACCGAGCCAGGCGGTAGGAGTACCCAGGACGCTTCTCGCTTTGAGCCCATAACCATCAGACGACC
```

TGAGATATAGATGAGAGTCAGTAACACCGAGCAGTCGCTGCGCTAGTCAACACGAGTGAATAACAGTCCGAACAGTG
TGTAGTGGCATTGTGCTAAAGCGTCGGGGTCATTGTAATTGGTCAAGATACTGGCTGCCTTNNNNNNNNNNNNNNNN
NNNNCGTTGCCGAAGCGATTGAACTCCGCTGGATCTATGCCATC

ErrorRateCaller.R

The central R script used to identify UMI barcodes, rebin them, group them, and call errors on them.

```
args<-commandArgs(trailingOnly=TRUE)
inputFileID<-args[1]
ncores<-as.numeric(args[2])-1

minQuorum=12;
cat("Loading libraries ...\n")
#Import libraries
invisible(sapply(c("dplyr","binaryLogic","stringr","parallel","data.table","p
bapply","compiler","stringi","seqTools","fst","triebeard","gtools","Rcpp","pu
rrr","seqinr","permutations","ggplot2","plyr","zeallot"),function(x){library(
x,quietly=T,character.only = T)}))

cat("Loading methods ...\n")
#For Parsing FASTQ Header Files (Not Used)
pboptions(type="txt")
parseFastQHeader<-function(header)
{
  header<-unlist(strsplit(gsub("/",":",gsub(c("#"),":",header)),":"))
  header[-1]
}

#For Parsing Cigar Strings to Cigar Character Vectors
expandCigar<-cmpfun(function(cigar){rep(stri_split_charclass(cigar,patter="[0
-9]",omit_empty = T)[[1]],as.numeric(stri_split_charclass(cigar,patter="[D,H,
I,M,N,P,S,X,=]",omit_empty = T)[[1]]))})

#get number of soft-clipped bases in a read from a cigar string
findSClipNum<-cmpfun(function(cigar){if(stri_split_charclass(cigar,patter="[0
-9]",omit_empty = T)[[1]][1]=="S"){as.numeric(stri_split_charclass(cigar,patt
er="[D,H,I,M,N,P,S,X,=]",omit_empty = T)[[1]][1])}else{0}})

#Import the reference sequence
reference_seq<-paste(read.fasta(file="QRGX_reference.fa")[[1]],collapse = "")

#For extracting a barcode from each read
extractSequenceAtLocus<-cmpfun(function(seq,cigar,pos,sClipNum,barcodeStart=7
55,barcodeEnd=774,offset=0,modStart=0,modStop=0)
{
  pos=as.numeric(pos)-sClipNum
  if(stri_detect_charclass(pattern="[DI]",str=cigar)){
    cigarex<-expandCigar(cigar)
```

```
    x=pos
    for(i in 1:length(cigarex))
    {
      if(barcodeStart==x){modStart=i}
      if(barcodeEnd==x){modStop=i;break()}
      if(cigarex[i]=="I"){}
      else{if(cigarex[i]=="D"){x<-x+2}
      else{x<-x+1}}
    }
    stri_sub(seq,modStart,modStop)
  }
  else{
    stri_sub(seq,barcodeStart-pos+1,barcodeEnd-pos+1)}
})

#import useful Rcpp-accelerated functions
sourceCpp("QRGX_Processing_Functions.cpp")

#Chunk generator for given parameters, used for apply-class functions
chunker<-function(nrow,chunkSize)
{
  numChunks<-floor(nrow/chunkSize)
  chunkMat<-data.frame(start=((0:numChunks)*chunkSize)+1,stop=((1:(numChunks+
1))*chunkSize))
  if(nrow%%chunkSize!=0){chunkMat[numChunks+1,2]<-nrow}else{chunkMat<-chunkMa
t[-nrow(chunkMat),]}
  chunkMat
}

#Useful method for Phred Lookups
phredVec<-readRDS("phredVec.rds")
phredProbLookup<-cmpfun(function(x) sapply(lapply(x,function(x){phredVec[strs
plit(x,split="")[[1]]]}),prod))

#build all possible sequences from degenerate barcode sequence
seq_permutations<-cmpfun(function(barcode,hamming_distance){
  possibles=barcode
  for(i in 1:hamming_distance){
    possibles<-stri_replace_first_charclass(possibles,"[N]",rep(c("A","T","C"
,"G"),each=length(possibles)))
  }
  unique(possibles)
})

#build all possible sequences within a given hamming distance from a specific
barcode based on bad phred scores
generate_barcode_bin<-cmpfun(function(barcode,qual,hamming_distance)
{
  if(hamming_distance!=0){
    sub<-unname(stri_locate_all_charclass(qual,"[!\"#$%\\&'()*+,]",merge=F)[[
```

```
1]][,1])
    if(!is.na(sub[1])){
      hamming_distance<-ifelse(hamming_distance>length(sub),length(sub),hammi
ng_distance)
      sub_mat<-combinations(length(sub),hamming_distance,sub)
      seq_permutations(apply(sub_mat,1,function(x){stri_sub_all_replace(barco
de,x,x,replacement = "N")}),hamming_distance=hamming_distance)
    }else{barcode}
  }else{barcode}
})
cat("Building the cluster ...\n")
#Now Make the Cluster
cl<-makeCluster(ncores)
clusterEvalQ(cl,{library(stringi)})
clusterEvalQ(cl,{library(Rcpp)})
clusterExport(cl,c("phredVec"))
clusterExport(cl,c("as.binary","extractSequenceAtLocus","expandCigar"))
clusterEvalQ(cl,{sourceCpp("QRGX_Processing_Functions.cpp")});

#Import the SAM file from the RAID
samColClasses<-c("character","numeric","character","numeric","numeric","chara
cter","character","numeric","numeric","character","character",rep("character"
,12))
samfile<-fread(input=paste(c("SAM Files/QRGX",inputFileID,".sam"),
                           collapse=""),
               sep="\t",
               skip=3,
               header=T,
               colClasses = samColClasses,showProgress = T,
               select=c(1,4,2,6,10,11,9),
               nThread = ncores,
               fill = T)

#set column names of samfile object
colnames(samfile)<-c("qname","pos","flag","cigar","seq","qual","tlen")

cat("Parsing SAM file flags ...\n")
#Parsing SAM file flags
flagMat<-pbsapply(samfile$flag,function(x){asBinary(x)[c(1,5,7)]},cl=cl)
rownames(flagMat)<-c("paired","reverse_strand","first")

cat("Assigning strand directionality to read table ...\n")
#Assigning strand directionality information to samfile object
samfile[,reverse_strand:=flagMat["reverse_strand",]]
samfile[,first:=flagMat["first",]]
samfile<-samfile[tlen!=0&flagMat["paired",],]
tlenMean<-median(abs(samfile$tlen))
samfile<-samfile[which((abs(samfile$tlen)>(tlenMean-200))&(abs(samfile$tlen)<
(tlenMean+200))),] #select reads with an estimated template length of the med
ian ±200 nt
```

```r
samfile[,template:=(samfile$reverse_strand!=samfile$first)] #identify templat
e vs polymerized strands
rm(flagMat) #remove transient SAM file flag object

#Add soft-clip information to samfile object
sClipNums<-pbsapply(samfile$cigar,findSClipNum,cl=cl)
samfile[,sClipNum:=sClipNums]
rm(sClipNums)

cat("Identifying barcodes ...\n")
#Identifying barcodes
chunkMat<-chunker(nrow(samfile),chunkSize = 7.5E5) #split samfile into chunks
barcodes<-unlist(pbapply(MARGIN=1,chunkMat,function(y){parApply(X=samfile[y[1
]:y[2],c("tlen","seq","cigar","pos","sClipNum")],MARGIN=1,FUN=function(x){
  ifelse(as.numeric(x[1])<0,extractSequenceAtLocus(seq=x[2],cigar = x[3],pos=
x[4],sClipNum=as.numeric(x[5])),NA)
},cl=cl)})) #get barcode for each read in samfile
cat(paste(c("Found ",length(unique(barcodes))," in ",nrow(samfile)/2," total 
barcodes."),collapse=""))
samfile[,barcode:=barcodes] #add barcode information to samfile
rm(barcodes) #remove barcode object from memory

cat("Identifying barcode quality ... \n")
barcodeQuals<-unlist(pbapply(MARGIN=1,chunkMat,function(y){parApply(X=samfile
[y[1]:y[2],c("tlen","qual","cigar","pos","sClipNum")],MARGIN=1,FUN=function(x
){
  ifelse(as.numeric(x[1])<0,extractSequenceAtLocus(seq=x[2],cigar = x[3],pos=
x[4],sClipNum=as.numeric(x[5])),NA)
},cl=cl)})) #run the same script on PHRED score strings instead, pulling the 
substrings of each barcode
samfile[,barcodeQual:=barcodeQuals] # add this info to the samfile object fro
m memory

cat("Calculating barcode probability of error ... \n")
pErBars<-1-pbsapply(samfile$barcodeQual,function(x){ifelse(!is.na(x),prod(1-p
hredVec[strsplit(x,split="")[[1]]]),NA)},cl=cl) #calculate the probability th
at a barcode is in error based on its phred scores
samfile[,pErBars:=pErBars] #add this information to the samfile
rm(pErBars,barcodeQuals) #remove barcode quality and probability objects from
memory

cat("Copying barcodes to forward oriented reads ... \n")
#Copying barcodes to forward oriented reads
setorder(samfile,"qname")
stopifnot(identical(samfile[samfile$first,"qname"],samfile[!samfile$first,"qn
ame"]))
tlenMat<-cbind(samfile$tlen[seq(1,nrow(samfile)-1,by=2)],samfile$tlen[seq(2,n
row(samfile),by=2)])
badRows<-which(rowSums(tlenMat)!=0) # make sure template lengths for forwards
and reverse reads are the same, record rows where this is not true
```

```
rm(tlenMat) #remove tlenMat from memory

badRows<-c(badRows*2,badRows*2-1) # get bad row indices of samfile from bad r
ows of tlenMat
if(!is_empty(badRows)){ badRows<-badRows[order(badRows)];samfile<-samfile[-ba
dRows,]} #if badrows is not empty, order an remove badrows
samfile[tlen>0,c("barcode","pErBars","barcodeQual")]<-samfile[tlen<0,c("barco
de","pErBars","barcodeQual")] #copy barcode information to forward oriented r
eads
samfile<-samfile[nchar(samfile$barcode)==20,] #select reads which have a 20-n
t barcode only, removing poorly aligned ones
rm(badRows) #remove badrows from memory

cat("Binning barcodes to determine barcode-ID ... \n")
#Binning barcodes to determine barcode-ID
barcodes<-unique(samfile$barcode) #get unique barcodes
barcode_trie<-trie(barcodes,1:length(barcodes)) #build radix tree from unique
barcodes
chunkMat<-chunker(nrow(samfile),chunkSize = 5E5) # generate chunks for barcod
e identification
pb <- txtProgressBar(min = 0, max = nrow(chunkMat), style = 3) #initialize pr
ogress bar
IDList<-list()
for(i in 1:nrow(chunkMat)){
  IDList[[i]]<-longest_match(trie=barcode_trie, to_match = samfile$barcode[ch
unkMat[i,1]:chunkMat[i,2]])
  setTxtProgressBar(pb, i)
} #bin reads based on common barcodes
samfile[,barcodeID:=unlist(IDList)] # assign a numerical barcode bin ID to ea
ch read
rm(IDList,barcode_trie,chunkMat) #remove intermediate objects from memory

cat("Taking out the garbage ... \n")
gc() #take out the trash!

cat("Identifying possible alternate bins for low-frequency barcodes ...\n")
#Rebin barcodes
hamming_distance=1; #set max hamming distance parameter

#export some things to the cluster
clusterExport(cl,c("generate_barcode_bin","seq_permutations","hamming_distanc
e"))
clusterEvalQ(cl,{library(gtools)})
clusterEvalQ(cl,{library(stringi)})

#Frequencies of each barcode:
barcodeFreqs<-table(samfile$barcodeID)

#Identify 'bad barcodes', those which occur exactly once or have a probabilit
y of error greater than 5%.
```

```
badBars<-intersect(as.numeric(names(barcodeFreqs[barcodeFreqs<=2])),samfile[which(samfile$pErBars>=0.05),"barcodeID"][[1]])

#identify target 'good barcodes', those which greater than 10 occurences.
goodBars<-as.numeric(names(barcodeFreqs[barcodeFreqs>=10]))
print(paste("found ",length(badBars)," poorly mapped barcodes, fixing now!"))

#identify alternate barcodes which could possibly be the true identity of each bad barcode exactly x hamming distance away.
badBarAlternateList<-pbapply(samfile[(samfile$barcodeID%in%badBars)&(samfile$first),c("barcode","barcodeQual")],MARGIN=1,cl=cl,function(x,hamming_dist){
  tryCatch(generate_barcode_bin(x[1],x[2],hamming_distance),error=NA)
},hamming_dist=hamming_distance)

#Make the lookup tree for the good barcodes.
barcode_trie<-trie(barcodes[goodBars],goodBars)

cat("Re-binning bad barcodes to correct destiations ...\n")

#rebin the bad barcodes to their correct destinations if possible
badBarMapList<-pblapply(X=badBarAlternateList,function(x){
  na.omit(longest_match(trie=barcode_trie, to_match = x))})
nSubBars<-sapply(badBarMapList,length)
exclusionList<-unlist(badBarMapList[nSubBars>1])
badBarMapList<-unlist(badBarMapList[nSubBars==1])
badBars<-badBars[nSubBars==1]

#keep track of reads which were rebinned
reBinnedCol=samfile$barcodeID%in%badBars
samfile[,rebinned:=reBinnedCol]
samfile[,"barcodeID"]<-replace(samfile$barcodeID,which(reBinnedCol),rep(badBarMapList,each=2))
print(paste("Rebinned ",length(badBars)," bad barcodes"))
rm(badBars,badBarMapList,badBarAlternateList,barcode_trie,reBinnedCol)
newFreqs<-table(samfile$barcodeID)

#update barcode frequency table
barcodeFreqs<-newFreqs[order(newFreqs,decreasing=T)]
barcodeFreqs<-barcodeFreqs[order(barcodeFreqs,decreasing=T)]
barcodeFreqs<-barcodeFreqs[barcodeFreqs<500]
goodBars<-as.numeric(names(barcodeFreqs[barcodeFreqs>=minQuorum])) #identify good barcode bins as being those which have more than 12 member reads (6 mate pairs))
rm(newFreqs,barcodeFreqs) #remove barcode frequency objects from memory

rowCutoffThreshold=12 #if a set of template or polymerized reads at a given locus has more than x members, then do not compute probabilities of error above this threshold
consensusThreshold=0.5 #minimum consensus threshold
```

```r
baseVec1<-c(1,2, 3, 5, 7) #template prime number IDs
baseVec2<-c(1,11,13,17,19) #polymerized prime number IDs
names(baseVec1)<-c("A","T","C","G","I") #template prime number ID vector name
s
names(baseVec2)<-c("A","T","C","G","D") #polymerized prime number ID vector n
ames
MSIDVec<-factor(c("M","S","I","D"),levels=c("M","S","I","D")) # MSID= match,
substitution, insertion, deletion
reference_seq_vec=reference_seq%>%toupper%>%strsplit(.,"")%>%.[[1]] #generate
a vector of single characters from the reference sequence

#load in required libraries into the cluster
invisible(clusterEvalQ(cl,{library(dplyr)}))
invisible(clusterEvalQ(cl,{library(stringi)%>%invisible}))
invisible(clusterEvalQ(cl,{library(Rcpp)%>%invisible}))
invisible(clusterEvalQ(cl,{library(data.table)%>%invisible}))
invisible(clusterEvalQ(cl,{library(plyr)%>%invisible}))
invisible(clusterEvalQ(cl,{library(zeallot)%>%invisible}))
invisible(clusterExport(cl,"reference_seq_vec"))

#generate all possible combinations of mate pairs for a group of 6-12, store
in a list.
combinationMatList<-pblapply((minQuorum/2):rowCutoffThreshold,function(x){exp
and.grid(replicate(x, 0:1, simplify = FALSE))})
combinationMatList<-pblapply(1:length(combinationMatList),function(x){combina
tionMatList[[x]][which(rowSums(combinationMatList[[x]])>(x+minQuorum/2-1)/2),
]%>%as.matrix})

#central function for generating error statistics for a given barcode bin
generateConsensusMat<-cmpfun(function(seq,qual,pos,cigar,template,pErBars){
  cigarex<-lapply(cigar,expandCigar) # generate cigar expansions for each cig
ar string
  startPos<-sapply(cigarex,function(x){which(x!="S")[1]-1}) # identify starti
ng positions for each sequence
  c(seqMat,qualMat,insLocs,consensusVecTemplate,tempLens,consensusVecPolymeri
zed,polyLens,delLocsP,correct_reference_bases,commonCols)%<-%consensusCoreLoo
p(seq,qual,pos,cigarex,startPos,any(cigarex%>%unlist=="I"),template,minQuorum
/2,consensusThreshold,reference_seq_vec) #returns sequence and quality matrix
alignments, consensus base at each locus, quora for template and polymerized
reads, as well as insertion&deletinon data
  if(sum(commonCols)>1){ #if polymerized and template consensus sequences hav
e common loci, then continue
    seqMat=seqMat[,commonCols,drop=F] #subset by common columns
    qualMat<-1-(matrix(1-phredVec[qualMat[,commonCols]],nrow(qualMat))*(1-pEr
Bars)) #convert phred quality matrix to an error probability matrix
    data.table(MSIDVec[(((names(consensusVecTemplate[commonCols])!=names(cons
ensusVecPolymerized[commonCols]))>(insLocs[commonCols]|delLocsP[commonCols]))
+insLocs[commonCols]*2+delLocsP[commonCols]*3)+1],
               1-getPE2(seqMat[template,],qualMat[template,],names(consensusV
ecTemplate[commonCols]),rowCutoffThreshold,minQuorum,insLocs[commonCols])*get
```

```
PE2(seqMat[!template,],qualMat[!template,],names(consensusVecPolymerized[comm
onCols]),rowCutoffThreshold,minQuorum,delLocsP[commonCols]),
               as.numeric(colnames(seqMat)),
               baseVec1[names(consensusVecTemplate[commonCols])]*baseVec2[nam
es(consensusVecPolymerized[commonCols])],
               tempLens[commonCols],
               polyLens[commonCols],
               consensusVecTemplate[commonCols],
               consensusVecPolymerized[commonCols],
               correct_reference_bases[commonCols]) #build data table summari
zing all the data, including type of error, PE calculations, and quality cont
rol parameters for that base
  }
  })

#export more methods and objects to the cluster, as well as sourcing Rcpp met
hods
clusterExport(cl,c("expandCigar","minQuorum","rowCutoffThreshold","consensusT
hreshold"))%>%invisible
clusterExport(cl,c("generateConsensusMat","combinationMatList","MSIDVec","phr
edVec","between","baseVec1","baseVec2","minQuorum"))%>%invisible
clusterEvalQ(cl,{library(purrr);library(partitions);sourceCpp("/home/rstudio/
getPE.cpp");sourceCpp("consensusCoreLoop.cpp");})%>%invisible

#build chunks for processing the samfile
chunkMat<-chunker(length(goodBars),5E4)

cat("Splitting data based on barcodes...\n")
#split samfile into a list of data.tables based on common UMI elements
samSplitList<-flatten(pbapply(chunkMat,1,function(x){split(samfile[which(barc
odeID%in%goodBars[x[1]:x[2]]),],by="barcodeID",)}))
rm(samfile) #remove samfile to save on memory
gc() #collect garbage
samSplitList<-samSplitList[between(pbsapply(samSplitList,function(x){ifelse(m
in(x$pos)<=754,sum(x$template)/nrow(x),0)},cl=cl),0.125,8)] #remove entries f
rom the list which do not have sequences intersecting the template, i.e. remo
ves artifacts, also remove list entries which have a temp:polymerized strand
ratio of <1:8 or >8:1

cat("Calculating base-wise accuracy expectations ...\n")
errorTable<-pblapply(samSplitList,function(x){generateConsensusMat(x$seq,x$qu
al,x$pos,x$cigar,x$template,x$pErBars)},cl=cl) #call the generateConsensusMat
function on each barcode bin in the list to generate the errorTable
errorTable<-rbindlist(l,use.names = F,idcol = T) #combine each list entry int
o one data.table, keep a numerical inded indicating the list entry from which
each row originated
colnames(l)<-c("barcodeID","error","PE","locus","errorID","quorum_T","quorum_
P","cons_T","cons_P","temp_ref_match") #set column names for errorTable
errorTable[,E:=abs((error=="S")-PE)] #define E (expected error) for a substit
ution
```

```r
cat("Writing to FST...\n") #write errorTable to FST.
write_fst(as.data.frame(errorTable),paste(c("Error Tables/QRGX",inputFileID,"_finalErrorTable.fst"),collapse=""))

stopCluster(cl)
```

QRGX_Processing_Functions.cpp

Supplementary C++ functions for UMI barcode identification to be called in R using Rcpp.

```cpp
#include <Rcpp.h>
using namespace Rcpp;

//Convert Integer to Binary, accelerated using Rcpp
//[[Rcpp::export]]
LogicalVector asBinary(int n)
{
  // array to store binary number
  LogicalVector bN(8);
  int a = 0;
  while (n > 0) {
    bN[a] = bool (n % 2);
    n = n / 2;
    a++;
  }
  return bN;
}

//Accelerated Rcpp Function for Extracting Barcodes within extractSequenceAtLocus()
//[[Rcpp::export]]
std::string barcodeExtractorLoop(CharacterVector cigarex,int pos,std::string seq,int barcodeStart=755,int barcodeEnd=774,NumericVector modStartStop=NumericVector::create(0,0))
{
  for(int i=0;i<cigarex.size();i++)
  {
    if(barcodeStart==pos){modStartStop[1]=i;}
    if(barcodeEnd==pos){modStartStop[2]=i;break;}
    if(Rcpp::as<char>(cigarex[i])=='I'){}
    else if(Rcpp::as<char>(cigarex[i])=='D'){pos=pos+2;}
    else{pos=pos+1;}
  }
  return seq.substr(modStartStop[1],modStartStop[2]-modStartStop[1]+1);
}
```

consensusCoreLoop.cpp

C++ functions to be called in R using Rcpp for processing alignment data and determining consensus read sequences as well as quality control and insertion/deletion calling.

```
#include <Rcpp.h>
using namespace Rcpp;

// [[Rcpp::plugins("cpp11")]]

// [[Rcpp::export]]
List consensusCoreLoop(const CharacterVector &seq,const CharacterVector &qual
,const NumericVector &pos,const List &cigarex,const NumericVector &startPos,b
ool insPresent,NumericVector temp,int minQuorum,double consensusThreshold,Cha
racterVector reference_seq_vec, int maxPos=754)
{
  int minPos=min(pos); //minimum position on the template
  CharacterVector cig; //cigar vector placeholder
  NumericVector insLocs; //vector of number of inserted loci per read
  int insOffset; //running insertion offset moving through the read
  int insertions=0; //placeholder for number of insertions on a given read
  IntegerVector col_names=Rcpp::seq(minPos, maxPos); //column names of seqMat
, qualMat, equal to the locus of a given column in the template
  if(insPresent){ //only execute insertion calculations if insertions are pre
sent in this read set
    for(int i=0;i<cigarex.size();i++){ //iterate i over the number of items i
n the expanded cigar list (cigarex)
      insertions=0; //set insertions to 0
      cig=as<CharacterVector>(cigarex[i]); //set cig = the cigar expansion lo
cated at index i in the cigarex list
      for(int j=startPos[i];j<cig.length();j++) //iterate j over the individu
al characters in the cigar vector
      {
       if((as<char>(cig[j]))=='I'){insLocs.push_back(j+pos[i]-minPos-insertio
ns-startPos[i]);insertions++;} //if the entry of cig[j] is an insertions, add
this locus to the insertion loci vector, and do positional correction for the
start locus of the read, and the number of extand insertions in the read matr
ix
       if((j+pos[i]-startPos[i]-insertions+1)>(maxPos)){break;} // if the loc
us being evaluated is past the maximum position (754), then stop
      }
    }
    insLocs=sort_unique(insLocs); //get unique insertion loci
    insertions=insLocs.size(); //number of insertions from insLocs
    for(int i=0;i<insertions;i++) //iterate over insertions
    {
      col_names.insert(insLocs[i]+i,insLocs[i]+minPos); //posotional correcti
on. i.e. an insertion at locus 1 makes a second insertion at locus 3 actually
occur at locus 4 because of the added base upstream
      insLocs[i]=insLocs[i]+i; // commit this correction
```

```
    }
  }
  CharacterMatrix seqMat(seq.size(),maxPos-minPos+1+insertions); //generate a matrix to hold the aligned read sequences for error calling and consensus read generation, correcting for insertions
  CharacterMatrix qualMat(seq.size(),maxPos-minPos+1+insertions);//generate a matrix to hold the aligned read quality vectors for error calling and consensus read generation, correcting for insertions
  LogicalVector n(seqMat.ncol()); //logical vector for which columns are insertions, and not the next locus
  for(int i:insLocs){n[i]=true;} //build this vector.
  colnames(seqMat)=as<CharacterVector>(col_names); //set column names for seqMat
  colnames(qualMat)=as<CharacterVector>(col_names); //set column names for qualMat
  std::string seqVec; //placeholder std::string for holding a given base sequence
  std::string qualVec; //placeholder std::string for holding a given quality sequence
  int x; //initialize counter x
  int delOffset; //running deletion offset moving through the read
  for(int i=0;i<seqMat.nrow();i++) //iterate over rows of seqMat, aka iterate over each read in the UMI bin
  {
    x=startPos[i]; //x= start position of the read in question at row i
    cig=as<CharacterVector>(cigarex[i]); //cigar = character vector of the cigar vector at cigarex list entry i
    seqVec=as<std::string>(seq[i]); //seqVec = base sequence string at seq vector index i
    qualVec=as<std::string>(qual[i]); //qualVec = quality sequence string at qual vector index i
    delOffset=0; //deletion offset = 0
    for(int j=pos[i]-minPos;j<seqMat.ncol();j++) //iterate j over loci in the sequence of read i, adjust for read start position
    {
        if(x<seq[i].size()){
          if(as<bool>(any(j==(insLocs)))){ // if j is an insertion locus
            if(as<char>(cig[x+delOffset])=='I'){ //if, after accounting for deletion offset, is the cigar for this read at this locus an insertion
              seqMat(i,j)=seqVec.substr(x,1); //assign the character at locus x in seqVec to seqMat[i,j]
              qualMat(i,j)=qualVec.substr(x,1); //assign the character at locus x in qualVec to seqMat[i,j]
              x++; //increase x counter
            }
          }
          else if(as<char>(cig[x+delOffset])=='D'){ //if the cigar character at this index, after compensating for deletions, is a deletion
            seqMat(i,j)="D"; //add the placeholder character D to seqMat to signify a deleted base
```

```
          delOffset++; //add one to the deletion offset
        }
        else{
          seqMat(i,j)=seqVec.substr(x,1); //assign the character at locus x
in seqVec to seqMat[i,j]
          qualMat(i,j)=qualVec.substr(x,1); //assign the character at locus
x in qualVec to seqMat[i,j]
          x++;
        }
      }
    }
  }
  int length=0; //placeholder length variable
  std::vector<NumericVector> consensus(2); //a vector of two numeric vectors,
which explain the consensus at each base for the template and polymerized str
and respectively
  std::vector<std::vector<int>> atcg(2, std::vector<int>(5,0)); //two counter
s for consensus determination, one for template and one for polymerized
  std::string bases[5]={"A","T","C","G","D"}; //possible entries in seqMat wh
ich are counted, remember we want to know if a deleted base is the consensus
as well, so it acts just like A,T,C, or G here
  std::vector<int> nblanks={0,0}; //number of blank spaces for a given column
of seqMat (for both template and polymerized), not counted in consensus
  std::vector<int> maxIndex={0,0}; // maximum index of the bases vector for a
given locus for both temp and poly
  IntegerMatrix quorumMat(2,seqMat.ncol()); //matrix holding transient consen
sus information
  LogicalMatrix delMat(2,seqMat.ncol()); //matrix holding deletion informatio
n
  std::string name; //consensus vector name placeholder
  for(int i=0;i<seqMat.ncol();i++) //iterate over each column of seqMat
  {
    std::fill(maxIndex.begin(),maxIndex.end(),0); //set maxindex to 0,0
    for(int j=0;j<2;++j){std::fill(atcg[j].begin(),atcg[j].end(),0);} //set a
tcg vectors to 0 for both temp and poly
    std::fill(nblanks.begin(),nblanks.end(),0); //set nblanks vector to 0
    for(int j=0;j<seqMat.nrow();j++) //iterate j over each row of seqMat colu
mn i
    {
      switch(as<char>(seqMat(j,i))) //switch depending on the base present at
seqMat[j,i]
      {
      case 'A':{atcg[temp[j]][0]++;break;} // if A, at 1 to the atcg vector o
f either template or polymerized depending on whether this row is either of t
he two
      case 'T':{atcg[temp[j]][1]++;break;} // if T...
      case 'C':{atcg[temp[j]][2]++;break;} // if C...
      case 'G':{atcg[temp[j]][3]++;break;} // if G...
      case 'D':{atcg[temp[j]][4]++;break;} // if D...
      default:{nblanks[temp[j]]++;break;}  // if this is a blank, then at it
```

```
to nblanks for either temp or poly
      }
    }
    for(int k=0;k<2;k++) //iterate k over 0 and 1 for temp (0) and poly (1) c
onsensus determination
    {
      maxIndex[k]=std::distance(atcg[k].begin(),std::max_element(atcg[k].begi
n(), atcg[k].end())); //figure out which index of atcg[k] has the maximal val
ue
      if(maxIndex[k]==4){quorumMat(k,i)=std::abs(sum(temp-1+k))-nblanks[k];}e
lse{quorumMat(k,i)=std::abs(sum(temp-1+k))-nblanks[k]-atcg[k][4];} //deal wit
h a maxIndex being a deletion
      if((k==1)&as<bool>(any(i==insLocs))){name="I";}else{name=bases[maxIndex
[k]];} //set name variable to either an I or the base which is present depend
ing on if it is in insLocs
      if(quorumMat(k,i)>=minQuorum) //if quorum of reads is reached based on 
minQuorum
      {
        if(maxIndex[k]==4){delMat(k,i)=true;} //add deletion data to delmat i
f applicable
        consensus[k].push_back(((double)atcg[k][maxIndex[k]]/quorumMat(k,i)),
name); //add consensus of this locus to the consensus vector, make its name t
he identity of the consensus base, or deletion.
      }
      else{consensus[k].push_back(0,name);} //if no quorum reached, then make 
consensus 0 because we dont want it.
    }
  }
  //this section determines if the consensus base of the template is the same
or different than the QRGX reference sequence.
  LogicalVector correctRef(consensus[1].length());
  insOffset=0;
  CharacterVector cvT=consensus[1].names();
  for(int i=0;i<consensus[1].length();i++)
  {
    if((bool)n[i]|(bool)delMat(1,i))
    {
      correctRef[i]=NA_LOGICAL;
      insOffset++;
    }
    else if(cvT[i]==reference_seq_vec[minPos+i-1-insOffset]){correctRef[i]=tr
ue;}
  }
   return List::create(seqMat,qualMat,n,consensus[1],quorumMat(1,_),consensus
[0],quorumMat(0,_),delMat(0,_),correctRef,(((consensus[1]>consensusThreshold)
!=n)>delMat(1,_))&(consensus[0]>consensusThreshold)); //return relevant infor
mation regarding sequence and quality matrices, quora, and consenses
}
```

getPE.cpp

C++ functions to be called in R using Rcpp for calculating PE values.

```
#include <RcppArmadillo.h>
using namespace Rcpp;

// [[Rcpp::depends(RcppArmadillo)]]
// This initialization function reads in the combinationMatList R object from
the global environment and converts it into a vector of Rcpp-armadillo vector
s
std::vector<arma::mat> generateCML(){
  std::vector<arma::mat> cml;
  List combMatList=Environment::global_env()["combinationMatList"];
  for(int i=0;i<combMatList.size();i++){cml.push_back(as<arma::mat>(combMatLi
st[i]));}
  return cml;
}
//set cml = combinationMatList from R
std::vector<arma::mat> cml=generateCML();

//product function... gets the product of a given Rcpp NumericVector
double product(NumericVector vec){return std::accumulate(vec.begin(),vec.end(
),1.0,std::multiplies<double>());}

//gets the sum of all row-based products of the combination matrix and q-scor
e vector for a given locus
// [[Rcpp::export]]
double rowProdSum2(int whichMat,const arma::rowvec &inputQ){return sum(arma::
prod(arma::abs(cml[whichMat].each_row()-inputQ),1));}

//analogous to the R order function
// [[Rcpp::export]]
IntegerVector order2_min(NumericVector vec,int min){
  NumericVector vec2=clone(vec);
  IntegerVector retVec(vec2.size());
  for(int i=0;i<vec2.length();++i){retVec[i]=which_min(vec2);vec2[retVec[i]]=
R_PosInf;}
  return retVec[Range(0,std::min(min-1,int(vec2.length()-1)))];
}
//remove blanks ("") from a vector
CharacterVector blank_omit(StringVector vec)
{
  for(int i=vec.size()-1;i>=0;--i){if(vec[i]==""){vec.erase(i);}}
  return vec;
}
//analogous to na.omit in R
NumericVector na_omit2(NumericVector vec)
{
  for(int i=vec.size()-1;i>=0;--i){if(R_IsNA(vec[i])){vec.erase(i);}}
```

```
  return vec;
}

//Get PE values from a sequence matrix given phred quality matrix data, and c
onsensus data
// [[Rcpp::export]]
NumericVector getPE2(CharacterMatrix seqMat,NumericMatrix qualMat,CharacterVe
ctor consensusSeq,const int &rowCutoffThreshold,int minQuorum,LogicalVector e
xcLocs)
{
  CharacterVector seqVec;
  NumericVector qualVec(0);
  NumericVector peVec=NumericVector(seqMat.ncol())+1;
  NumericVector ord;
  int i=0;
  for(int i=0;i<seqMat.ncol();i++)
  {
    if((bool)(excLocs[i])!=true)
    {
       qualVec=na_omit2(qualMat(_,i));
       ord=order2_min(qualVec,rowCutoffThreshold);
       seqVec=blank_omit(seqMat(_,i))[ord];
       qualVec=qualVec[ord];
       for(int j=0;j<seqVec.size();j++){if(seqVec[j]!=consensusSeq[i]){qualVe
c[j]=1-qualVec[j];}}
       peVec[i]=rowProdSum2(seqVec.size()-(minQuorum/2),as<arma::rowvec>(qual
Vec));
    }
  }
  return peVec;
}
```

CalledFSTCombiner.R

R script for performing meta-analysis on all the individual error tables called from each .bam file. This includes code for plotting and statistical analysis of the results.

```
#import necessary libraries
library(fst)
library(data.table)
library(dplyr)
library(plyr)
library(tidyr)
library(ggalt)
library(pbapply)
library(seqinr)
library(dqrng)
library(tibble)
library(ggpval)
library(ggpubr)
```

```r
library(rstatix)
library(Rmisc)


#import reference QRGX sequence
reference_seq<-paste(read.fasta(file="QRGX_reference.fa")[[1]],collapse = "")
#import reference_seq from FASTA
reference_seq_vec=reference_seq%>%toupper%>%strsplit(.,"")%>%.[[1]] #split re
ference_seq string and make upper case
reference_seq_multi=data.table(base=reference_seq_vec,locus=1:length(referenc
e_seq_vec))[1:754,] #define table from reference_seq, organize by locus
reference_seq_multi[,triNuc:=c(sapply(locus[1:752],function(x){substr(referen
ce_seq,x,x+2)%>%toupper}),c(NA,NA))] #iterate over reference_seq by dinucleot
ide
reference_seq_multi[,diNuc:=c(sapply(locus[1:753],function(x){substr(referenc
e_seq,x,x+1)%>%toupper}),NA)] #iterate over reference_seq by trinucleotide

#some externally-sourced functions for statistical analyses/plotting
addPval=function(plot,pairs,heightOffset=.9,...){
  add_pval(plot, pairs = pairs,heights=cumprod(c(max(ggplot_build(plot)$layou
t$panel_params[[1]]$y.range),rep(heightOffset,length(pairs)-1))))
}
get_legend<-function(myggplot){
  tmp <- ggplot_gtable(ggplot_build(myggplot))
  leg <- which(sapply(tmp$grobs, function(x) x$name) == "guide-box")
  legend <- tmp$grobs[[leg]]
  return(legend)
}

#miscellaneous functions
zero_omit=function(x){x[x!=0]}
nan_omit=function(x){x[!is.nan(x)]}
gc_content=function(x){nchar(gsub("[G,C]+","",x))/nchar(x)}
isPurine=Vectorize(function(x){toupper(substr(x,1,1))%in%c("A","G")})

#colour schemes for use
{
cbp1 <- c("#999999", "#E69F00", "#56B4E9", "#009E73",
                  "#F0E442", "#0072B2", "#D55E00", "#CC79A7")[c(6,7,3,5)]
Tol_bright <- c('#EE6677', '#228833', '#4477AA', '#CCBB44', '#66CCEE', '#AA33
77', '#BBBBBB')[c(3,6,5,1)]
Tol_light <- c('#BBCC33', '#AAAA00', '#77AADD', '#EE8866', '#EEDD88', '#FFAAB
B', '#99DDFF', '#44BB99', '#DDDDDD')[c(3,2,7,1)]
Tol_light2<- c('#BBCC33', '#AAAA00', '#77AADD', '#EE8866', '#EEDD88', '#FFAAB
B', '#99DDFF', '#44BB99', '#DDDDDD')[c(3,4,7,6)]
}

#Various LUTs
groupIDs=factor(rep(1:4,each=3)) #groupID look-up-table (LUT)
baseIDs=outer(c(1,2,3,5,7),c(1,11,13,17,19))%>%as.vector()%>%unique #baseID L
```

```
UT
errorIDs=outer(c(1,2,3,5),c(1,11,13,17))%>%as.vector()%>%unique%>%.[-c(1,6,11
,16)] #errorID LUT
originErrorBases=rep(c("A","T","C","G"),4)%>%.[-c(1,6,11,16)] #get template b
ase from errorID
destinationBases=rep(c("A","T","C","G"),each=4) #get substituted base from er
rorID
baseIDSymbols=paste(rep(c("A","T","C","G"),by=4),"→",destinationBases) #get f
igure substitution symbol from baseID
errorIDSymbols=baseIDSymbols%>%.[-c(1,6,11,16)] #get figure substitution symb
ol from errorID
diNucs=apply(expand.grid(rep(list(c('A', 'G', 'T', 'C')), 2)),1,function(x){p
aste(x,collapse = "")}) #all possible dinucleotides
diNucLUT=cbind(expand.grid(rep(list(diNucs), 2)),1:256)%>%as.data.table #dinu
cleotide lookup table
names(diNucLUT)=c("template","destination","ID") # rename dinucleotide lookup
table
locusDiNucIDMap=match(reference_seq_multi$diNuc,diNucs) #identify dinucleotid
es at each template locus
gravity=factor(rep(c("1G","μG"),each=2),levels=c("1G","μG")) #get gravity fro
m groupID
exo=factor(rep(c("Klenow (exo+)","Klenow (exo-)"),2),levels=c("Klenow (exo+)"
, "Klenow (exo-)")) #get exo from groupID
niceNames=factor(c("1G+","1G-","μG+","μG-"),levels=c("1G+","1G-","μG+","μG-")
) # get sample names for figure plotting from groupID
purinePyrimidine=setNames(rep(c("purine","pyrimidine"),2),c("A","T","G","C"))
# purine/pyrimidine from a DNA base

#Import masterErrorTable from FST and format for analysis
cat("combining list into data.table")
pblapply(c(1:12),function(x){read_fst(paste(c("Error Tables/QRGX",x,"_finalEr
rorTable.fst"),collapse = ""),as.data.table = T)})%>%rbindlist(.,use.names =
F,idcol = T)->masterErrorTable
colnames(masterErrorTable)[1]<-"replicateID" #Fix replicateID naming conventi
on
masterErrorTable[,barcodeID:=as.numeric(barcodeID)] #Fix barcodeID classifica
tion
masterErrorTable[,barcodeID:=as.numeric(.GRP),by=.(replicateID,barcodeID)] #M
ake barcodeIDs unique across test groups
masterErrorTable[,groupID:=groupIDs[replicateID]] #define groupID as the test
condition, replicateID is the specific replicate
masterErrorTable=masterErrorTable[temp_ref_match|is.na(temp_ref_match),] #rem
ove bases where the template does not match the reference sequence
badBars=masterErrorTable[,.(errors=sum(error=="S")),by=barcodeID][order(error
s),][errors>3,barcodeID] #identify bad barcodes, those that have more than 3
errors per template
masterErrorTable<-masterErrorTable[which(!barcodeID%in%badBars),] #remove the
bad barcodes
masterErrorTable=masterErrorTable[cons_P==1,] #Keep only bases where there is
absolute consensus in the polymerized strand
```

```
gc() #collect the trash

#Process error calculations
PEcutoffTable=masterErrorTable[,.(cutoff=10^(-10:0),error=sapply(10^(-10:0),function(x){.SD[PE<=x,mean(E)]}),.N),by=.(groupID,replicateID,locus)] #obtain process error
PEcutoffTable[,base:=reference_seq_vec[locus]] #define the base from each locus on the reference sequence
PEcutoffTableNoLoc=PEcutoffTable[locus>575,median(error),by=.(replicateID,groupID,cutoff,base)] #subset loci we want to interrogate and obtain median error, grouped by replicateID, groupID, PE cuttoff and base

#plot the PE density information
PEdensity=ggplot(masterErrorTable[,sample(PE,10000),by=.(niceNames[groupID],replicateID)])+
  theme_bw()+
  scale_color_manual(values=Tol_bright)+
  scale_fill_manual(values=Tol_bright)+
  geom_density(aes(x=-log10(V1),color=niceNames,group=replicateID))+xlab(bquote("-log"[10]~.("(process error)")))+theme(legend.position = "none",legend.title = element_blank(),legend.spacing.x = unit(.5, 'cm'))+
  geom_vline(aes(xintercept=5),linetype="dashed",color="black")+coord_cartesian(ylim=c(0,.5))+
  theme(text=element_text(size=16))

#Process error plotting, sectioned by base
PEPlot=ggplot(PEcutoffTableNoLoc)+
  theme_bw()+
  scale_color_manual(values=Tol_bright)+
  scale_fill_manual(values=Tol_bright)+
        geom_line(aes(x=-log10(cutoff),y=log10(V1),color=niceNames[groupID],group=replicateID))+
        facet_wrap(~base)+
        xlab(bquote("-log"[10]~.("(process error cut-off)")))+
        ylab(bquote("log"[10]~.("(error rate)")))+
        theme(legend.position = "bottom",legend.title = element_blank(),text=element_text(size=16),legend.spacing.x = unit(.1, 'cm'))+
        geom_vline(aes(xintercept=5),linetype="dashed",color="black")

#Combine two process error plots
gridExtra::grid.arrange(PEdensity+labs(title="a)"),PEPlot+labs(title="b)"),nrow=2)

#Based on visual inspection, keep PEs <1E-5
masterErrorTable=masterErrorTable[PE<1E-5,]

#Plot polymerization product lengths for each test group
tlen2=masterErrorTable[,min(locus),by=.(replicateID,groupID,barcodeID)]
TlenPlot=ggplot(tlen2[,sample(V1,10000),by=.(groupID,replicateID)])+
  theme_bw()+
```

```
  scale_color_manual(values=Tol_bright)+
  scale_fill_manual(values=Tol_bright)+
  geom_jitter(aes(y=754-V1,x=replicateID,color=niceNames[groupID],group=replicateID),size=0.05,alpha=0.1,width=0.42,height=5)+
  geom_boxplot(aes(y=754-V1,x=replicateID,fill=niceNames[groupID],group=replicateID),size=0.5,width=0.5,outlier.alpha = 0)+
  ylim(50,425)+
  xlab("sample")+
  ylab("polymerization product length (bp)")+
  theme(text = element_text(size=16),legend.position="bottom",legend.spacing.x = unit(.25, 'cm'),legend.title = element_blank(),axis.title.x=element_blank(),axis.text.x=element_blank(),axis.ticks.x=element_blank())

locusLimits=575:750 #Define limits of the loci which we want to interrogate

#build summary table sectioned by base substitution.
sumTableSectioned=masterErrorTable[locus%in%locusLimits&!error%in%c("D","I"),.(.N,errorID=errorIDs,conversion=errorIDSymbols,base=reference_seq_vec[locus],destination=rep(c("A","T","C","G"),each=3),Nerror=sapply(errorIDs,function(x){.SD[errorID==x,sum(E)]})),by=.(locus,replicateID,groupID)][originErrorBases[match(errorID,errorIDs)]==base,]
sumTableSectioned[,errorRate:=Nerror/N] #define error rate
sumTableSectioned[,gravity:=gravity[groupID]] #get gravity from groupID
sumTableSectioned[,exo:=exo[groupID]] #get exonuclease identity from groupID
sumTableSectioned[,niceName:=niceNames[groupID]] #get niceName from groupID
sumTableSectioned[,triNuc:=sapply(locus,function(x){substr(toupper(reference_seq),x,x+2)})] #get trinucleotide at this base
sumTableSectioned[,diNuc:=sapply(locus,function(x){substr(toupper(reference_seq),x,x+1)})] #get dinucleotide at this base

#plot error rate by base substitution identity, grouping by template base
p1=sumTableSectioned[((errorID==3&errorRate<5E-4)|errorID!=3)&errorRate!=0][,.(errorRate=c(errorRate,rep(mean(errorRate),3-.N))),by=.(niceName,exo,base,locus,conversion)]%>%
  ggplot(aes(x=conversion,y=log10(errorRate)))+
  theme_bw()+scale_fill_manual(values=Tol_bright)+
  stat_boxplot(aes(fill=niceName),geom="errorbar",width=.5,position = position_dodge(.75))+
  geom_boxplot(aes(fill=niceName),outlier.alpha = 0)+
  ylim(-6,-2)+
  theme(axis.text.x = element_text(angle = 90, hjust = 1),legend.position="bottom",title = element_text("template base"),text = element_text(size=16),legend.spacing.x = unit(.5, 'cm'))+
  ylab(bquote("log"[10]~.("(error rate)")))+
  xlab("base substitution")+
  labs(fill = "")+
  stat_pvalue_manual(
    cbind(sumTableSectioned[((errorID==3&errorRate<5E-4)|errorID!=3)&errorRate!=0][,.(errorRate=c(errorRate,rep(mean(errorRate),3-.N))),by=.(niceName,exo,base,locus,conversion)]%>%group_by(conversion,exo,base)%>%wilcox_test(formula
```

```
=errorRate~niceName)%>%adjust_pvalue(method="fdr")%>%add_x_position(x="conver
sion")%>%mutate(xmin=rep(xmin[1:6],4),xmax=rep(xmax[1:6],4)),y.position=rep(c
(-5,-4,-3.75,-3,-5,-3.75,-3.5,-3.75,-3.75,-4.25,-3.25,-4.25),each=2)),
    label="p.adj.signif",step.increase = 0.1,step.group.by = "conversion",hid
e.ns = T,size=4.5)+
  facet_wrap(~base,scale="free_x",nrow=1)

#bootstrap median difference in error rate between gravitational conditions t
o obtain effect sizes and standard-deviation
bootstrappedBasewiseDifferentialTable=sumTableSectioned[((errorID==3&errorRat
e<5E-4)|errorID!=3)&errorRate!=0][,.(errorRate=c(errorRate,rep(mean(errorRate
),3-.N))),by=.(niceName,exo,base,locus,conversion,gravity,destination)][,repl
icate(1000,{median(.SD[gravity=="µG",errorRate]%>%sample(.,length(.),replace
= T))/median(.SD[gravity=="1G",errorRate]%>%sample(.,length(.),replace = T))-
1})*100,by=.(exo,conversion,destination)][!is.infinite(V1)][,summarySE(.SD,me
asurevar = "V1"),by=.(conversion,exo,destination)]
#plot bootstrap data, grouping by substituted base
p2=ggplot(bootstrappedBasewiseDifferentialTable)+
  theme_bw()+scale_fill_manual(values=Tol_bright)+
  geom_bar(aes(x=conversion,y=V1,fill=exo),stat="identity",position="dodge",c
olor="black")+
  geom_errorbar(aes(ymin=V1-sd,ymax=V1+sd,x=conversion,group=exo),color="blac
k",position=position_dodge(.9),width=.4)+
  theme(axis.text.x = element_text(angle = 90, hjust = 1),legend.position="bo
ttom",legend.title = element_blank(),legend.spacing.x = unit(.5, 'cm'),text =
element_text(size=16))+
  facet_wrap(~destination,scales="free_x",nrow=1)+
  xlab("base substitution")+
  ylab("differential median error rate (µG vs. 1G) %")+
  stat_pvalue_manual(cbind(sumTableSectioned[((errorID==3&errorRate<5E-4)|err
orID!=3)&errorRate!=0][,.(errorRate=c(errorRate,rep(mean(errorRate),3-.N))),b
y=.(niceName,exo,base,locus,conversion,destination)]%>%group_by(conversion,ex
o,base,destination)%>%wilcox_test(formula=errorRate~niceName)%>%adjust_pvalue
(method="fdr")%>%add_x_position(x="conversion")%>%arrange(destination,convers
ion)%>%mutate(x=rep(rep(1:3,each=2),4)+rep(c(-1,1)*.225,12)),y.position=boots
trappedBasewiseDifferentialTable[order(destination,conversion),(V1+sd+50)%>%i
felse(.>50,.,50)]),
                    label="p.adj.signif",hide.ns = T,x="x",size=4.5)
gridExtra::grid.arrange(p1+labs(title="a)"),p2+labs(title="b)"),nrow=2) # gro
up these plots

#Combine individual substitution data at each locus to generate error rates b
y locus representative of all possible substitutions.
sumTableAll=sumTableSectioned[(errorID==3&errorRate<5E-4)|errorID!=3,.(errorR
ate=sum(Nerror)/N[1],N=N[1],Nerror=sum(Nerror)),by=.(locus,base,replicateID,g
roupID,gravity,diNuc,triNuc,exo,niceName)]

#plot a 25-period SMA of error rate moving across the template for each test
sample
p3=sumTableAll[,median(errorRate),by=.(locus,niceName)][order(niceName,-locus
```

```
)][,.(locus,errorRate=frollapply(V1,25,FUN=mean)),by=.(niceName)][,.(errorRat
e,locus=751-locus),by=niceName]%>%
  ggplot()+theme_bw()+scale_color_manual(values=Tol_bright)+
  geom_xspline(aes(x=locus,y=errorRate,color=niceName),spline_shape = 1)+
  ylab("error rate")+
  theme(legend.position = "bottom",legend.title = element_blank(),legend.spac
ing.x = unit(.25, 'cm'),text = element_text(size=16))+
  labs(title="a)")

#plot a 25-period SMA of gc-content moving across the template
p4=ggplot()+
  theme_bw()+
  geom_xspline(aes(x=locus,y=V2),spline_shape = 2,data=sumTableAll[replicateI
D==1,.(locus=751-locus,base),][order(locus)][,.(locus,frollapply(setNames(c(1
,2,3,4),c("A","T","C","G"))[base],25,function(x){gc_content(paste(c("A","T","
C","G")[x],collapse =""))}))])+
  ylab("gc content")+
  theme(text=element_text(size=16))+
  labs(title="b)")

#distribution of error rate increases (fold-change) from 1G to uG for each lo
cus
p5=sumTableAll[,median(errorRate),by=.(locus,niceName,exo)][order(niceName,-l
ocus)][,.(locus,errorRate=frollapply(V1,25,FUN=mean)),by=.(niceName,exo)][,.(
errorRate,locus=751-locus),by=.(niceName,exo)][,errorRate[2]/errorRate[1]-1,b
y=.(exo,locus)]%>%ggplot()+
  scale_color_manual(values=Tol_bright)+
  scale_fill_manual(values=Tol_bright)+
  geom_density(aes(y=..density..,x=V1,fill=exo,color=exo),adjust=2,alpha=.15)
+
  theme_bw()+
  xlab("error rate increase from 1G to µG (fold-change)")+
  theme(legend.title = element_blank(),legend.position = "bottom",legend.spac
ing.x = unit(.25,"cm"),text = element_text(size=16))+
  labs(title="c)")
cowplot::plot_grid(p3,p4,p5,align="v",ncol=1,rel_heights = c(1,0.4,1))

#Plot total and base-wise substitution error rate boxplots, as well as associ
ated statistics
p6=
  ggplot(data=cbind(sumTableAll,total="total"),mapping=aes(y=log10(errorRate)
,x=niceName))+
  theme_bw()+scale_fill_manual(values=Tol_bright)+
  stat_boxplot(geom="errorbar",width=.4,position = position_dodge(.75))+
  geom_boxplot(aes(fill=niceName),outlier.alpha = 0)+
  xlab("")+
  ylab(bquote("log"[10]~.("(error rate)")))+
  theme(axis.ticks.x=element_blank(),legend.text = element_text(size=16),axis
.text.x = element_blank(),legend.position = "top",legend.title = element_blan
k(),legend.spacing.x = unit(.25, 'cm'),text = element_text(size=16))+
```

```
  stat_pvalue_manual(
    sumTableAll[,.(errorRate=median(errorRate)),by=.(groupID,niceName,exo,gra
vity,locus)]%>%group_by(exo)%>%arrange(locus)%>%wilcox_test(errorRate~niceNam
e,p.adjust.method = "fdr",paired = T)%>%mutate(y.position=-3),
    label="p.adj",step.increase = 0.2,size=4.5)+
  stat_pvalue_manual(
    sumTableAll[,.(errorRate=median(errorRate)),by=.(groupID,niceName,exo,gra
vity,locus)]%>%group_by(exo)%>%arrange(locus)%>%wilcox_test(errorRate~niceNam
e,p.adjust.method = "fdr",paired = F)%>%mutate(y.position=-2.85),
    label="p.adj",step.increase = 0.2,size=4.5,color=Tol_bright[4],bracket.si
ze = 0)+
  labs(title="a)")+
  ylim(NA,-2)+
  facet_wrap(~total,strip.position = "bottom")

legend=get_legend(p6) #save legend for plotting later
p6=p6+theme(legend.position = "none") #remove legend from p8 plot

#Plot base-wise error rates and statistics
p7=ggplot(sumTableAll,mapping=aes(y=log10(errorRate),x=niceName))+
  theme_bw()+scale_fill_manual(values=Tol_bright)+
  stat_boxplot(geom="errorbar",width=.4,position = position_dodge(.75))+
  geom_boxplot(aes(fill=niceName),outlier.alpha = 0)+
  xlab("")+
  ylab("")+
  theme(axis.text.x = element_blank(),legend.position = "none",axis.ticks = e
lement_blank(),legend.title = element_blank(),legend.spacing.x = unit(.25, 'c
m'),text = element_text(size=16),axis.text.y = element_blank())+
  facet_wrap(~base,nrow=1,strip.position =  "bottom")+
  stat_pvalue_manual(
    sumTableAll[,.(errorRate=median(errorRate)),by=.(groupID,niceName,exo,bas
e,gravity,locus)]%>%arrange(locus)%>%group_by(exo,base)%>%wilcox_test(errorRa
te~niceName,paired=T)%>%arrange(exo,base)%>%mutate(p.adj=signif(p.adj,2),y.po
sition=rep(c(-3.75,-3,-3.5,-3.5),2)),label="p.adj",step.increase = 0.1,step.g
roup.by = "base",size=4.5)+
  labs(title="b)")+
  ylim(NA,-2)

#Combine plots p6 and p7
gridExtra::grid.arrange(p6,p7,legend,layout_matrix=rbind(matrix(rep(c(1,2,2,2
),15),nrow=15,byrow = T),3))

#Dinucleotide summary table
diNucTable=sumTableAll[order(locus),.(locus,diNuc,exo,base,base2=c(base[-1],N
A),error=(errorRate+lag(errorRate,1))),by=.(niceName,replicateID)][!is.na(err
or)&!is.na(base2)]

#Error rate boxplots for individual dinucleotides
p8=ggplot(diNucTable,mapping=aes(x=diNuc,y=log10(error)))+
  theme_bw()+scale_fill_manual(values=Tol_bright)+
```

```
  stat_boxplot(aes(fill=niceName),geom="errorbar",width=.4,position = positio
n_dodge(.75))+
  geom_boxplot(aes(fill=niceName),outlier.alpha = 0)+
  theme(legend.position = "bottom")+
  facet_wrap(~base,scales="free_x",nrow=1)+
  xlab("dinucleotide")+
  ylab(bquote("log"[10]~.("(error rate)")))+
  theme(legend.title = element_blank(),legend.spacing.x = unit(.25, 'cm'))+
  stat_pvalue_manual(cbind(diNucTable%>%group_by(base,exo,diNuc)%>%arrange(lo
cus)%>%wilcox_test(formula=error~niceName,paired=T)%>%adjust_pvalue(method="f
dr")%>%add_x_position(x="diNuc")%>%arrange(diNuc,base)%>%mutate(xmin=rep(xmin
[1:8],4),xmax=rep(xmax[1:8],4)),y.position=rep(c(-3.65,-3.3,0,0,-3.1,-3.1,-3.
15,-3.05,-3,-3.15,-3.3,-3.15,-3.1,-3.15,-3.15,-3.1),each=2)),step.increase =
0.1,hide.ns = T,label = "p.adj.signif",step.group.by = "diNuc")

#Trinucleotide summary table
triNucTable=sumTableAll[order(locus),.(locus,triNuc,base,base2=c(base[-(1)],N
A),base3=c(base[-(1:2)],NA,NA),error=(errorRate+lag(errorRate,1)+lag(errorRat
e,2))),by=.(niceName,replicateID,exo)][!is.na(error)&!is.na(base3)]

#Combine di- and tri-nucleotide error tables into one
diTriNucTable=rbind(diNucTable[,.(error=mean(error)),by=.(niceName,replicateI
D,diNuc,exo)][,.(niceName,exo,error,nLength="dinucleotide",nucleotide=diNuc)]
,triNucTable[,.(error=mean(error)),by=.(niceName,replicateID,triNuc,exo)][,.(
niceName,exo,error,nLength="trinucleotide",nucleotide=triNuc)])
p9=ggplot(diTriNucTable,aes(x=niceName,y=log10(error)))+
  theme_bw()+scale_fill_manual(values=Tol_bright)+
  stat_boxplot(aes(fill=niceName),geom="errorbar",width=.4,position = positio
n_dodge(.75))+
  geom_boxplot(aes(fill=niceName),outlier.alpha = 0)+xlab("sample")+
  theme(legend.title = element_blank(),axis.ticks = element_blank(),axis.text
.x = element_blank(),legend.spacing.x = unit(.25, 'cm'),legend.position = "bo
ttom")+
  xlab("nucleotide length")+
  ylab(bquote("log"[10]~.("(error rate)")))+
  ylim(-4.75,-2.5)+
  facet_wrap(~nLength,strip.position = "bottom")+
  stat_pvalue_manual(cbind(diTriNucTable%>%group_by(exo,nLength)%>%arrange(nu
cleotide)%>%wilcox_test(formula=error~niceName,paired=T)%>%adjust_pvalue(meth
od="fdr")%>%mutate(p.adj=signif(p.adj,digits = 2)),y.position=c(-3.25,-3,-3,-
2.75)),label="p.adj",size=4.5)

#combine di- and tri-nucleotide plots
gridExtra::grid.arrange(p9+labs(title="a)")+theme(legend.position = "none",ax
is.ticks.x=element_blank(),text = element_text(size=16)),p8+labs(title="b)")+
theme(text = element_text(size=16)),nrow=2)

#plot histogram of dinucleotides in the template
p10=diNucTable[replicateID==1,.N,by=.(diNuc,replicateID)]%>%ggplot()+
  theme_bw()+scale_fill_manual(values=Tol_bright)+
```

```
  geom_bar(aes(x=diNuc,y=N,fill=factor(replicateID)),stat="identity")+
  ylab("dinucleotides in template")+
  xlab("dinucleotide")+
  theme(axis.text.x = element_text(angle = 90, hjust = 1,vjust=0.5,size=8),le
gend.position = "none")

#plot histogram of trinucleotides in the template
p11=triNucTable[replicateID==1,.N,by=.(triNuc,replicateID)]%>%ggplot()+
  theme_bw()+scale_fill_manual(values=Tol_bright[2])+
  geom_bar(aes(x=triNuc,y=N,fill=factor(replicateID)),stat="identity")+
  ylab("trinucleotides in template")+
  xlab("trinucleotide")+
  theme(axis.text.x = element_text(angle = 90, hjust = 1,vjust=0.5,size=8),le
gend.position = "none")

#combine these two histograms
gridExtra::grid.arrange(p10,p11)

#Deletion summary table
delTable=masterErrorTable[error!="I"&locus%in%locusLimits,.(meanD=mean(error=
="D"),nD=sum(error=="D"),.N,base=reference_seq_vec[locus]),by=.(groupID,repli
cateID,locus)]
delTable[,gravity:=gravity[groupID]]
delTable[,exo:=exo[groupID]]
delTable[,niceName:=niceNames[groupID]]

# plot overall deletion data and conduct statistical tests
p12=ggplot(data=delTable%>%mutate(total="total"),mapping=aes(x=niceName,y=log
10(meanD)))+
  theme_bw()+scale_fill_manual(values=Tol_bright)+
  stat_boxplot(aes(fill=niceName),geom="errorbar",width=.4,position = positio
n_dodge(.75))+
  geom_boxplot(aes(fill=niceName),color="black",outlier.alpha = 0)+
  ylab(bquote("-log"[10]~.("(deletion rate)")))+
  xlab("")+
  theme(text = element_text(size=16),axis.ticks.x = element_blank(),legend.po
sition = "none",axis.text.x = element_blank())+
  facet_wrap(~total,strip.position = "bottom")+
  stat_pvalue_manual(delTable[meanD!=0,.(meanD=c(meanD,rep(mean(meanD),3-.N))
),by=.(niceName,locus,exo,base)]%>%group_by(exo)%>%wilcox_test(meanD~niceName
)%>%adjust_pvalue(method="fdr")%>%mutate(y.position=c(-4,-3.65)),label="p.adj
",size=4.5)+
  ylim(-6,-3.4)+
  labs(title="a)")

# plot base-wise deletion data
p13=ggplot(data=delTable[meanD!=0,.(meanD=c(meanD,rep(mean(meanD),3-.N))),by=
.(niceName,locus,exo,base)]%>%mutate(total="total"),mapping=aes(x=niceName,y=
log10(meanD)))+
  theme_bw()+scale_fill_manual(values=Tol_bright)+
```

```
  stat_boxplot(aes(fill=niceName),geom="errorbar",width=.4,position = position_dodge(.75))+
  geom_boxplot(aes(fill=niceName),color="black",outlier.alpha = 0)+
  ylab(bquote("-log"[10]~.("(deletion rate)")))+
  ylab("")+
  xlab("deleted base")+
  theme(text = element_text(size=16),axis.ticks = element_blank(), axis.text.y = element_blank(),legend.position = "none",axis.text.x = element_blank(),legend.title = element_blank(),legend.spacing.x = unit(.25, 'cm'))+
  facet_wrap(~base,nrow=1,strip.position = "bottom")+
  stat_pvalue_manual(delTable[meanD!=0,.(meanD=c(meanD,rep(mean(meanD),3-.N))),by=.(niceName,locus,exo,base)]%>%group_by(exo,base)%>%wilcox_test(meanD~niceName)%>%arrange(base,exo)%>%adjust_pvalue(method="fdr")%>%mutate(p.adj=signif(p.adj,digits=3),y.position=c(-4,-3.65,-4.75,-4.4,-4.25,-3.9,-5,-4.65)),label="p.adj",size=4.5)+
  ylim(-6,-3.4)+
  labs(title="b)")

#Combine deletion data plots
p14=gridExtra::grid.arrange(p12,p13,legend,layout_matrix=rbind(matrix(rep(c(1,2,2,2),10),nrow=10,byrow = T),3))

#bootstrap median difference in deletion rate between gravitational conditions
bootstrappedBasewiseDifferentialDeletionTable=delTable[meanD!=0,.(meanD=c(meanD,rep(mean(meanD),3-.N))),by=.(niceName,locus,exo,gravity,base)][,replicate(1000,{median(.SD[gravity=="µG",meanD]%>%sample(.,length(.),replace = T))/median(.SD[gravity=="1G",meanD]%>%sample(.,length(.),replace = T))-1})*100,by=.(exo,base)][!is.infinite(V1)][,summarySE(.SD,measurevar = "V1"),by=.(exo,base)]%>%mutate(purpyr=purinePyrimidine[base])
p15=
  ggplot(bootstrappedBasewiseDifferentialDeletionTable,aes(x=base,y=V1))+
  theme_bw()+scale_fill_manual(values=Tol_bright)+
  geom_bar(aes(fill=exo),stat="identity",position="dodge",color="black")+
  geom_errorbar(aes(ymin=V1-sd,ymax=V1+sd,x=base,group=exo),color="black",position=position_dodge(.9),width=.4)+
  theme(legend.position="bottom",legend.title = element_blank(),legend.spacing.x = unit(.5, 'cm'),text = element_text(size=16))+
  facet_wrap(~purpyr,scales="free_x",nrow=1,strip.position = "bottom")+
  xlab("deleted base")+
  ylab("differential median deletion rate (µG vs. 1G) %")+
  stat_pvalue_manual(delTable[meanD!=0,.(meanD=c(meanD,rep(mean(meanD),3-.N))),by=.(niceName,locus,exo,base)]%>%mutate(purpyr=purinePyrimidine[base])%>%group_by(exo,base,purpyr)%>%wilcox_test(meanD~niceName)%>%arrange(base,exo,purpyr)%>%adjust_pvalue(method="fdr")%>%mutate(p.adj=signif(p.adj,digits=3),x=rep(c(1,2),each=4)+rep(c(-1,1)*.225,4),y.position=bootstrappedBasewiseDifferentialDeletionTable[order(base,exo),(V1+sd+25)%>%ifelse(.>25,.,25)]),label="p.adj.signif",x="x",size=4.5)+
  ylim(NA,200)+
  labs(title="c)")
```

```r
#combine p14 and bootstrapped deletion plot
gridExtra::grid.arrange(p14,p15,nrow=2)

#function adapted from material at https://pcrfidelityestimator.neb.com/#!/he
lp
pcrCorCopies=function(amplicon_length=1000,polymerase_error_rate=1E-4,PCR_rep
lication_efficiency=1.8,cycles=30)
{
  bigP=1-(1-polymerase_error_rate)^amplicon_length
  total_bases=2*amplicon_length
  mutated_bases=0
  correct_bases=2*amplicon_length
  total_copies=2
  mutated_copies=0
  correct_copies=2

  FCCs=rep(1,cycles+1)

  for(cycle in 1:cycles)
  {
    total_bases=total_bases*PCR_replication_efficiency
    mutated_bases=mutated_bases*PCR_replication_efficiency+correct_bases*(PCR
_replication_efficiency-1)*polymerase_error_rate
    correct_bases=total_bases-mutated_bases
    total_copies=total_bases/amplicon_length
    mutated_copies=mutated_copies*PCR_replication_efficiency+correct_copies*(
PCR_replication_efficiency-1)*bigP
    correct_copies=total_copies-mutated_copies
    FCCs[cycle+1]=1-mutated_copies/total_copies
  }
  data.table(cycle=0:cycles,FCC=FCCs)
}

#generate PCR fidelity table
amp_lens=c(.5,1,2,5,10)
taq_fidelity=1.5E-4
uG_Offset=sumTableAll[,median(errorRate),by=groupID][,V1[4]/V1[2]]
PCR_table=data.table(niceName=c("Taq 1G","Taq µG"),errorRate=c(taq_fidelity,t
aq_fidelity*uG_Offset))[,lapply(amp_lens*1000,function(x){pcrCorCopies(amplic
on_length = x,polymerase_error_rate = errorRate)})%>%rbindlist(idcol="amplico
n_length")%>%mutate(amplicon_length=amp_lens[amplicon_length]%>%factor,FCC=FC
C*100),by=.(niceName)]

#Plot fraction of correct sequences over the course of 30 PCR cycles for a 1
Kb template
PCR_table[amplicon_length==1]%>%ggplot()+
  theme_bw()+
  scale_color_manual(values=Tol_bright)+
  geom_line(aes(x=cycle,y=FCC,color=niceName))+
```

```
  ylab("Correct PCR copies (%)")+
  xlab("PCR cycle")+
  xlim(0,30)+
  scale_y_log10()+
  geom_label(aes(x=c(29,29),y=V1*1.5,group=niceName,label=paste(signif(V1,3),
"%",sep="")),data=PCR_table[amplicon_length==1,last(FCC),by=niceName])+
  theme(legend.position = "bottom",legend.title = element_blank(),legend.spac
ing.x = unit(.25, 'cm'),text=element_text(size=16))
```
